# Discovery of High-Affinity PDGF-VEGFR Interactions: Redefining RTK Dynamics

**DOI:** 10.1038/s41598-017-16610-z

**Published:** 2017-11-27

**Authors:** Spencer B. Mamer, Si Chen, Jared C. Weddell, Alexandra Palasz, Ashley Wittenkeller, Manu Kumar, P. I. Imoukhuede

**Affiliations:** Department of Bioengineering, University of Illinois at Urbana-Champaign, Urbana, IL USA

## Abstract

Nearly all studies of angiogenesis have focused on uni-family ligand-receptor binding, e.g., VEGFs bind to VEGF receptors, PDGFs bind to PDGF receptors, etc. The discovery of VEGF-PDGFRs binding challenges this paradigm and calls for investigation of other ligand-receptor binding possibilities. We utilized surface plasmon resonance to identify and measure PDGF-to-VEGFR binding rates, establishing cut-offs for binding and non-binding interactions. We quantified the kinetics of the recent VEGF-A:PDGFRβ interaction for the first time with K_D_ = 340 pM. We discovered new PDGF:VEGFR2 interactions with PDGF-AA:R2 K_D_ = 530 nM, PDGF-AB:R2 K_D_ = 110 pM, PDGF-BB:R2 K_D_ = 40 nM, and PDGF-CC:R2 K_D_ = 70 pM. We computationally predict that cross-family PDGF binding could contribute up to 96% of VEGFR2 ligation in healthy conditions and in cancer. Together the identification, quantification, and simulation of these novel cross-family interactions posits new mechanisms for understanding anti-angiogenic drug resistance and presents an expanded role of growth factor signaling with significance in health and disease.

## Introduction

The vascular endothelial growth factor (VEGF)-VEGF receptor (VEGFR) signaling family has been extensively studied because it is the major regulator of microvascularization^1^. VEGF-targeted monoclonal antibodies have been developed to inhibit microvascular growth; and VEGF has also been applied to promote vascularization in engineered grafts, bioreactors, and tissue^2^. However, anti-angiogenic approaches have not yielded the promise of sustained vascular inhibition nor have pro-angiogenic approaches yielded stable blood vessel growth^3–7^. This is likely due to the fact that angiogenesis involves several signaling pathways, in addition to VEGF, representing a cross-family signaling complexity that cannot be captured by targeting one growth factor alone.

Increasing evidence suggests that a cross-family view of cell signaling offers promise in controlling angiogenesis. For example, VEGF-inhibition eventually results in anti-angiogenic resistance and one resistance mechanism involves the upregulation of ancillary axes, including: platelet derived growth factors (PDGFs)^8,9^, fibroblast growth factor, and angiopoietins^10^. Moreover, incorporating a cross-family view of angiogenesis into therapeutics has led to synergistic effects and improved blood flow, when dual-growth factor therapy is applied in pre-clinical ischemia models^11^, and improved wound healing, when dual-growth factors are coupled to biomaterials for tissue-engineering^12,13^. Together, these cross-family compensatory mechanisms and therapeutic advances suggests a need to shift from examining the VEGF-family alone toward directed exploration of combined growth factor signaling pathways—a cross-family paradigm.

**Figure 1:**
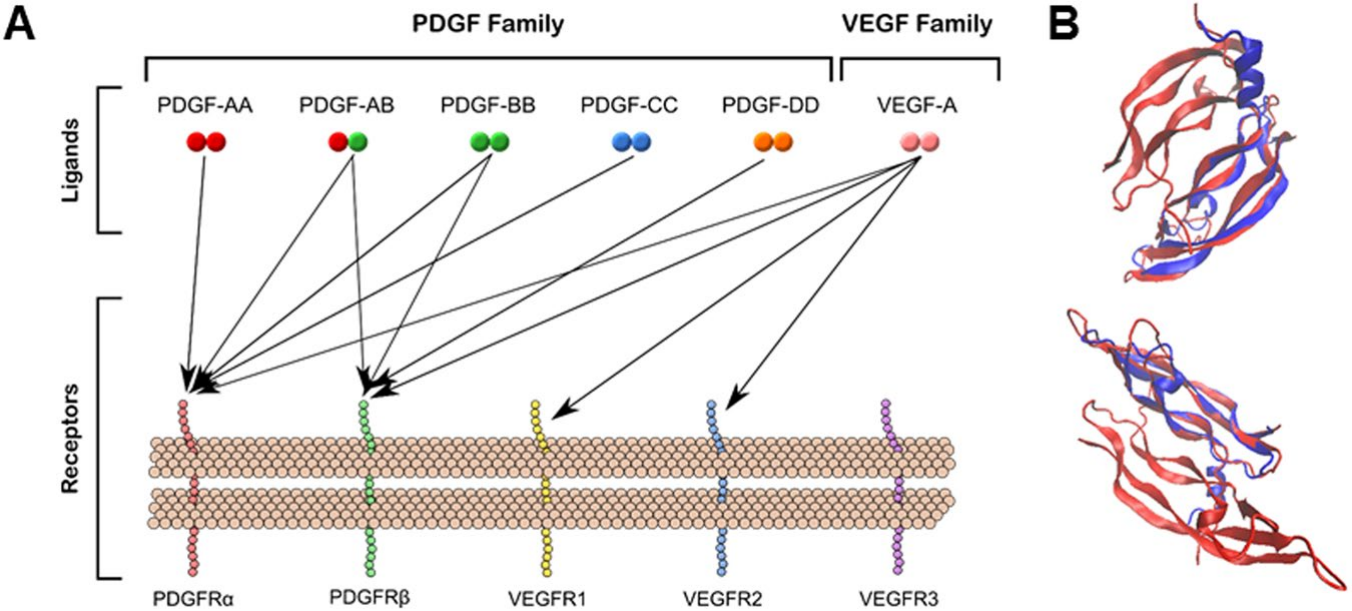
VEGF and PDGF ligand receptor interactions. **(A)** Schematic summarizes known receptors that bind VEGF-A and PDGFs. **(B)** Structural alignment of VEGF-A_165_ (blue, PDB ID: 2VPF) and PDGF-BB (red, PDB ID: 3MJG) proteins displayed from two rotational perspective.

The canonical angiogenesis philosophy involves uni-family ligand-receptor binding: VEGFs bind to VEGFRs, PDGFs bind to PDGFRs, and so on. This paradigm offers numerous activation schemes. For example, VEGF-A, -B, -C, -D, & placental growth factor and several isoforms produced via alternative splicing^14–19^ bind to selected VEGFRs: VEGFR1, -R2, -R3 and co-receptors: neuropilin-1 and neuropilin-2 leading to the angiogenic hallmarks of endothelial cell proliferation and migration to hypoxic regions^15,20^. PDGFs also contribute to vascular function. PDGF-AA, -BB, -AB, -CC, and –DD binding to corresponding PDGFRs (α and β) maintains and stabilizes endothelial tubes during development^21,22^, promotes endothelial cell proliferation^23^, induces vessel growth^24^ and regeneration^25^, and induces reperfusion after arterial blockage^26^. (A full schematic detailing the selective binding between these ligands and receptors is shown in Fig. 1).

Since VEGFs and PDGFs are both key regulators of angiogenesis, shifting the focus of angiogenesis away from a uni-family (VEGF-family alone) and towards a dual-family (VEGF and PDGF) focus would be a practical approach in exploring a cross-family angiogenesis framework. Indeed, one study of dose-dependent VEGF-A:PDGFR activation^27^ has provided an example of this new signaling paradigm. They found that VEGF-A directly binds both PDGF receptors and induce their phosphorylation in a dose-dependent fashion. However, additional cross-family interactions, such as PDGFs to VEGFRs and other VEGFs (VEGF-B, -C, and -D) to PDGFRs, remain unexplored.

There are several, compelling reasons why additional cross-family signaling interactions may occur. Structurally, PDGF and VEGF are similar, hailing from the cysteine knot superfamily^28^. The dimeric form of VEGF and PDGF shows significant alignment with a 1.8 Å root mean square difference^29^, despite the low VEGF—PDGF sequence homology (<20%). Furthermore, recent experimental studies established that VEGFR2 and PDGFRβ can form complexes on pericytes^30,31^, the mural cells that mediate vessel stability^32,33^. Therefore, measurement of possible cross-family interactions could reveal new angiogenic mechanisms.

Here we identify, measure, and simulate binding across the VEGF and PDGF families. We establish new standards for identifying and measuring new protein-protein interaction kinetics. We predict the significance of these cross-family binding interactions via computational modeling and present evidence that these novel interactions should significantly modulate angiogenic signaling, particularly in cancer. Overall, these cross-family interactions offer a shifted paradigm for understanding cell signaling with implications to angiogenesis, health, and disease.

## Results

### Validation of SPR protocol

**Figure 2:**
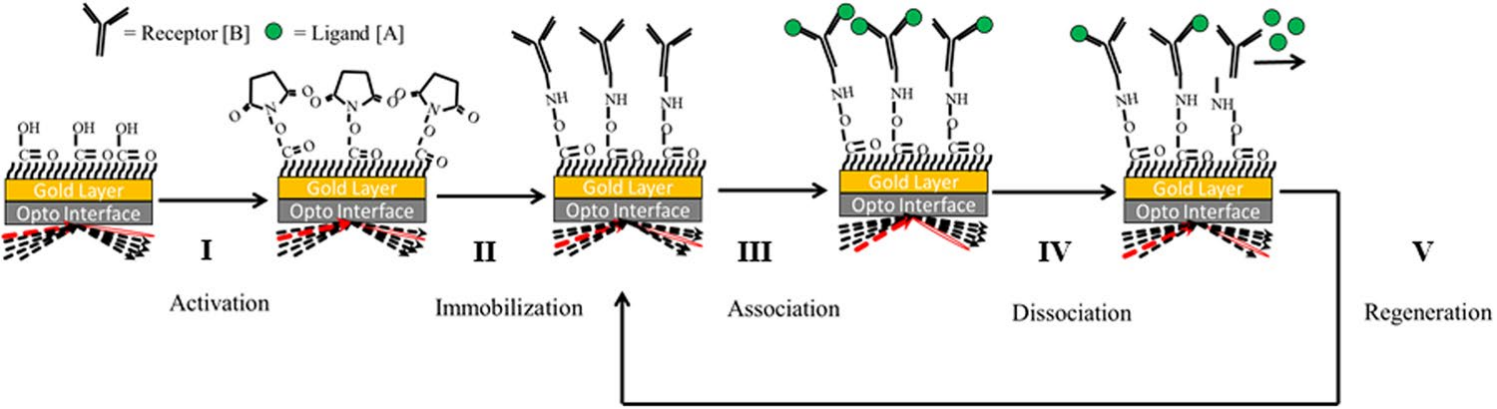
Surface plasmon resonance (SPR) workflow. Using a dextran-coated gold (CM5)-type sensor chip, SPR is performed as follows: (I) Carboxymethyl groups on the sensor surface are activated with NHS:EDC, enabling (II) amine-coupling between target (receptor or reference protein, Ang-4) and the dextran matrix semi-permanently. Such binding allows multiple kinetic cycles to be performed without requiring remobilization of the target. In a kinetic study cycle, (III) ligand is injected across the bound target; called the *association phase*, binding events are sensed and detected in real-time. (IV) Next running buffer (HBS-EP) alone is injected. In this *dissociation phase*, the instrument detects only the dissociation of ligand from the target. (V) A regeneration solution is injected to clear any remaining bound ligands before another kinetic study is performed.

We measured the kinetic rate constants between PDGF ligands and VEGF receptors using a surface plasmon resonance (SPR) assay. We injected the ligands at 40, 20 and 10 nM (process summarized in Fig. 2) and fit their association and dissociation curves using a 1:1 Langmuir binding model. To measure the accuracy of our kinetic analyses we applied a χ^2^-to-R_max_ ratio heuristic. For known interactions, a χ^2^-to-R_max_ value < 0.20, represents a well-established filter for high-quality fitting of kinetic parameters obtained via SPR^34–36^. The χ^2^-to-R_max_ ratio can be helpful in detecting new 1:1 binding interactions, because this value describes how well the obtained sensogram curves fit a 1:1 Langmuir interaction model. When the fitting noise (χ^2^) exceeds the maximal response (R_max_), the interaction cannot be said to follow the binding model. We therefore introduce a heuristic where a χ^2^-to-R_max_ ratio ≤ 1.0 is attributed to “true” interactions, and those >1.0 are described as non-1:1 Langmuir interactions resulting from non-specific binding.

To validate the χ^2^-to-R_max_ cut-off approach, we examined ligand:receptor pairs known to bind and non-interactions (i.e. pairs known not to bind). The χ^2^-to-R_max_ cut-off approach correctly identified well-established VEGF:VEGFR and PDGF:PDGFR ligand:receptor pairs as real interactions (χ^2^-to-R_max_ < 1.0), and accurately excluded those known to not interact (χ^2^-to-R_max_ > 1.0). Specifically, VEGF-A:VEGFR1 and VEGFR2, PDGF-AA:PDGFRa, PDGF-AB:PDGFRα, PDGF-BB:PDGFRs, and PDGF-CC:PDGFRa were correctly designated as real interactions, with χ^2^-to-R_max_ < 1.0 for each (Supplementary Table S1). The following known non-binding pairs were correctly excluded: VEGF-A:VEGFR3^37^ and PDGF-AA:PDGFRβ^38^, with χ^2^-to-R_max_ ratios 6.0- and 8.6-fold larger than the cut-off. The validation of the heuristic across 7 ligand:receptor pairs of the VEGFR and PDGFR families offers strong support for its extension to cross-family interactions within these families (Fig. 1 and Supplementary Table S1).

**Figure 3:**
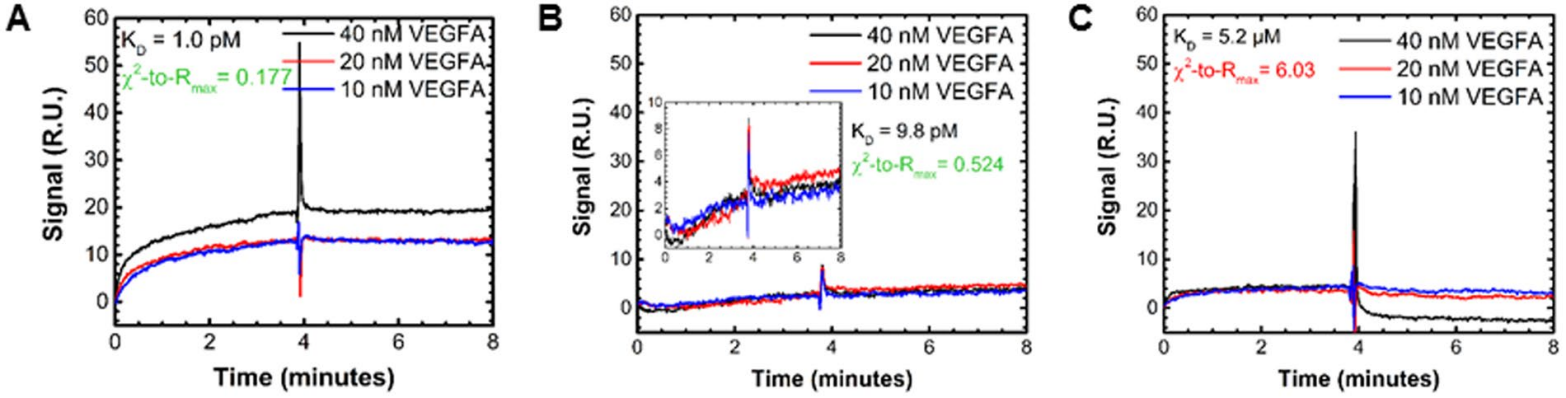
VEGF to VEGFR association and dissociation signal. BIAcore signal response kinetics studies of interactions between **(A)** VEGFA and immobilized VEGFR1 **(B)** VEGFA and VEGFR2 and **(C)** VEGFA and VEGFR3.

We further validated our experimental approach by reproducing previously measured binding affinities. The VEGF-A:VEGFR1 and VEGF-A:VEGFR2 binding affinities (Fig. 3A and Supplementary Table S2) were both within an order of magnitude of previous SPR measurements, K_D_ = 1 pM (Fig. 3A) versus a previous measured K_D_ = 7.5 pM^39^, and K_D_ = 9.8 pM (Fig. 3B.) versus 52 pM^40^, respectively.

### Establishing kinetic and affinity constants for canonical PDGF:PDGFR interactions

**Figure 4:**
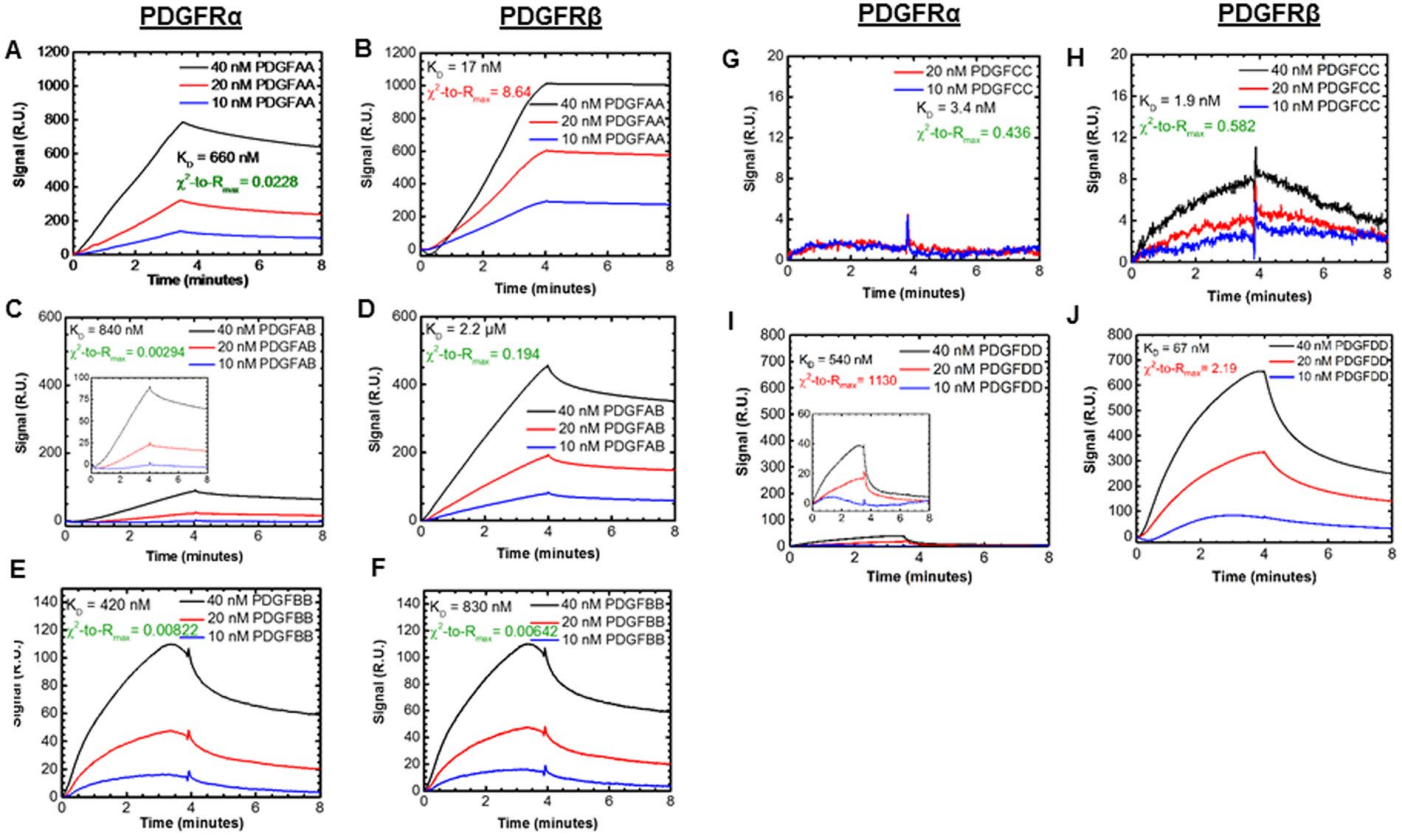
PDGF to PDGFR association and dissociation signal. BIAcore signal response kinetics studies of interactions between **(A)** PDGFAA and PDGFRα **(B)** PDGFAA and PDGFRβ **(C)** PDGFAB and PDGFRα **(D)** PDGFAB and PDGFRβ **(E)** PDGFBB and immobilized PDGFRα **(F)** PDGFBB and immobilized PDGFRβ **(G)** PDGFCC and immobilized PDGFRα **(H)** PDGFCC and immobilized PDGFRβ **(I)** PDGFDD and immobilized PDGFRα **(J)** PDGFDD and immobilized PDGFRβ.

We confirmed that the canonical understanding of PDGF:PDGFR holds (Fig. 1). More specifically, PDGF-AA binds only to PDGFRα (Fig. 4A,B)^41^; both PDGFRs bind PDGF-AB (Fig. 4C,D)^42^, -BB (Fig. 4E,F)^43^, and -CC (Fig. 4G,H)^44^; and PDGF-DD binds only to PDGFRβ (Fig. 4I,J)^42^. We then compared the obtained affinities, when possible, to previously reported affinities. Currently only PDGF-AA and –BB:PDGFR interactions have been reported via a study that did not use a reference protein cell (Supplementary Table S3). As described in the Materials and Methods, we used to reference protein to measure and account for non-specific binding effects that would affect our kinetics. All binding affinities values are listed exactly in Supplementary Table S2. We measured a PDGF-AA:PDGFRα affinity of the same order of magnitude as previously reported (measured K_D_ = 660 nM versus prior 134 nM^45^). We determined that PDGF-BB binds both PDGFRα and PDGFRβ with similar affinities K_D_ = 420 nM and 830 nM, respectively. While these measured affinities were lower than a previous report (K_D_ = 150 nM and 1.6 nM for PDGFRα and PDGFRβ, respectively) these previous studies did not use a reference protein^45^. We believe that this difference is critical: without a reference protein, these prior studies measure a weaker binding affinity due to an inflated non-specific binding reference measurement. Furthermore, the relative strength of binding –BB:Rα > —BB:Rβ, is observed in both our study and the work of others.

**Figure 5:**
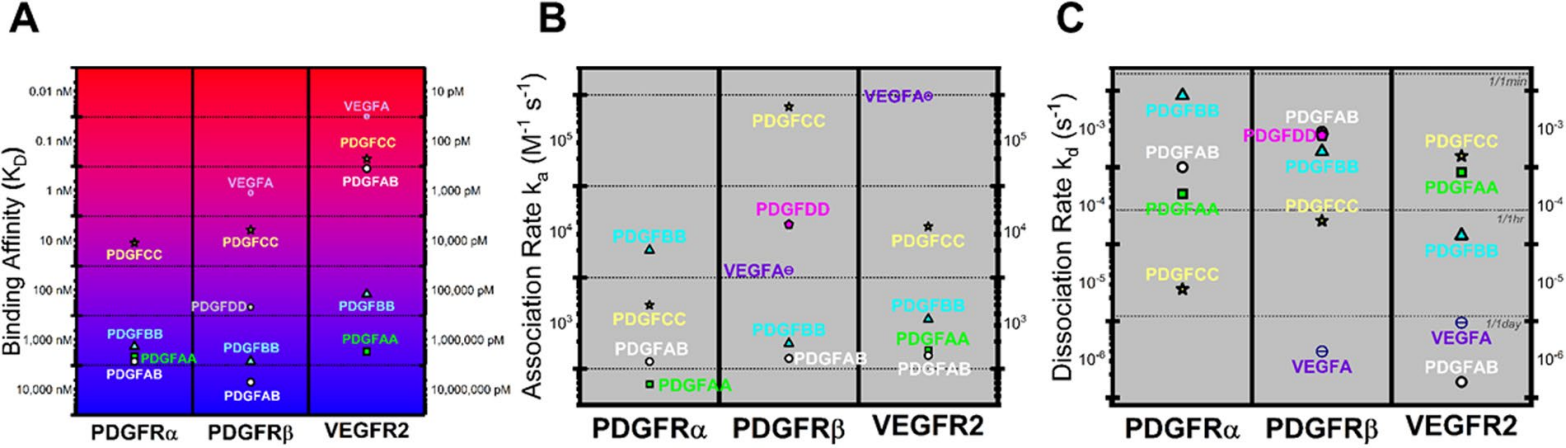
Binding kinetics and affinities for VEGF and PDGF interactions with PDGFRs and VEGFR2. **(A)** Binding affinity KD between each ligand and receptor. **(B)** Association rate (in M^−1^ s^−1^) and **(C)** dissociation rate (s^−1^).

We also characterized the binding rates and affinities for the canonical PDGF:PDGFR interactions lacking kinetic data (-AB, -CC, -DD:Rα and -AB, -CC, -DD:Rβ). We observed that PDGF-AB:PDGFRα binding affinity was ~2.6x stronger than PDGF-AB:PDGFRβ, with affinity constants of K_D_ = 840 nM versus K_d_ = 2,200 nM, respectively (Fig. 5A).We measured strong affinities between PDGF-CC:PDGFRα and PDGF-CC:PDGFRβ with K_D_ = 3.4 nM and 1.9 nM, (Fig. 5A) Finally, we observed that PDGF-DD binds PDGFRβ with moderate affinity, K_D_ = 67 nM (Fig. 5A). PDGF-DD:PDGFRβ has a 50-fold larger peak association response than its binding to PDGFRα, but its kinetic fitting for this interaction produced a χ^2^-to-R_max_ of 2.2 (Supplementary Table S1). But since it is >1.0, it displays high noise to signal, which we interpret as not true 1:1 Langmuir interactions.

### Novel cross-family PDGF:VEGFR2 interactions

**Figure 6:**
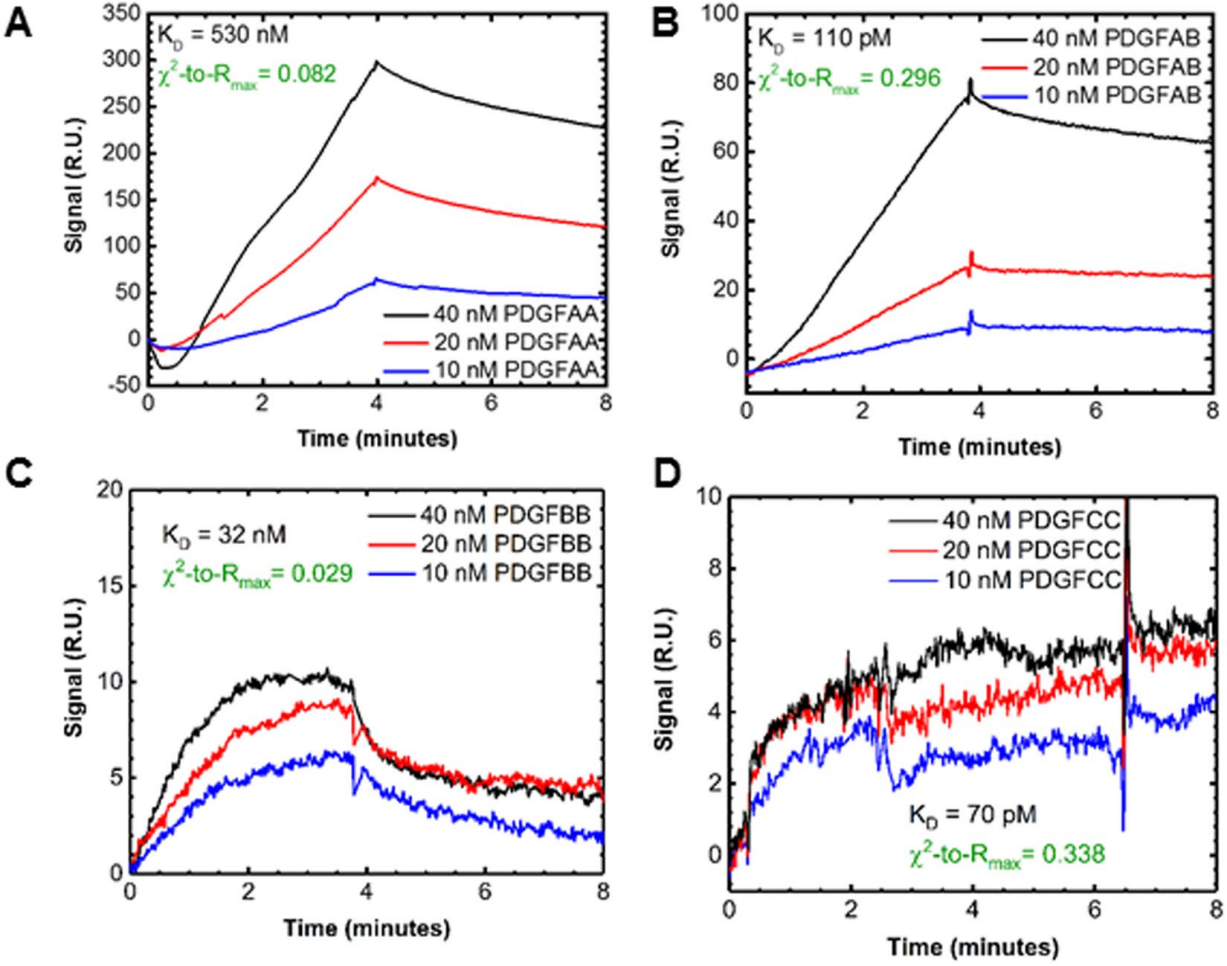
PDGF to VEGFR association and dissociation signal. BIAcore signal response kinetics studies of interactions between **(A)** PDGFAA and VEGFR2 **(B)** PDGFAB and VEGFR2 **(C)** PDGFBB and VEGFR2 and **(D)** PDGFCC and VEGFR2.

**Figure 7:**
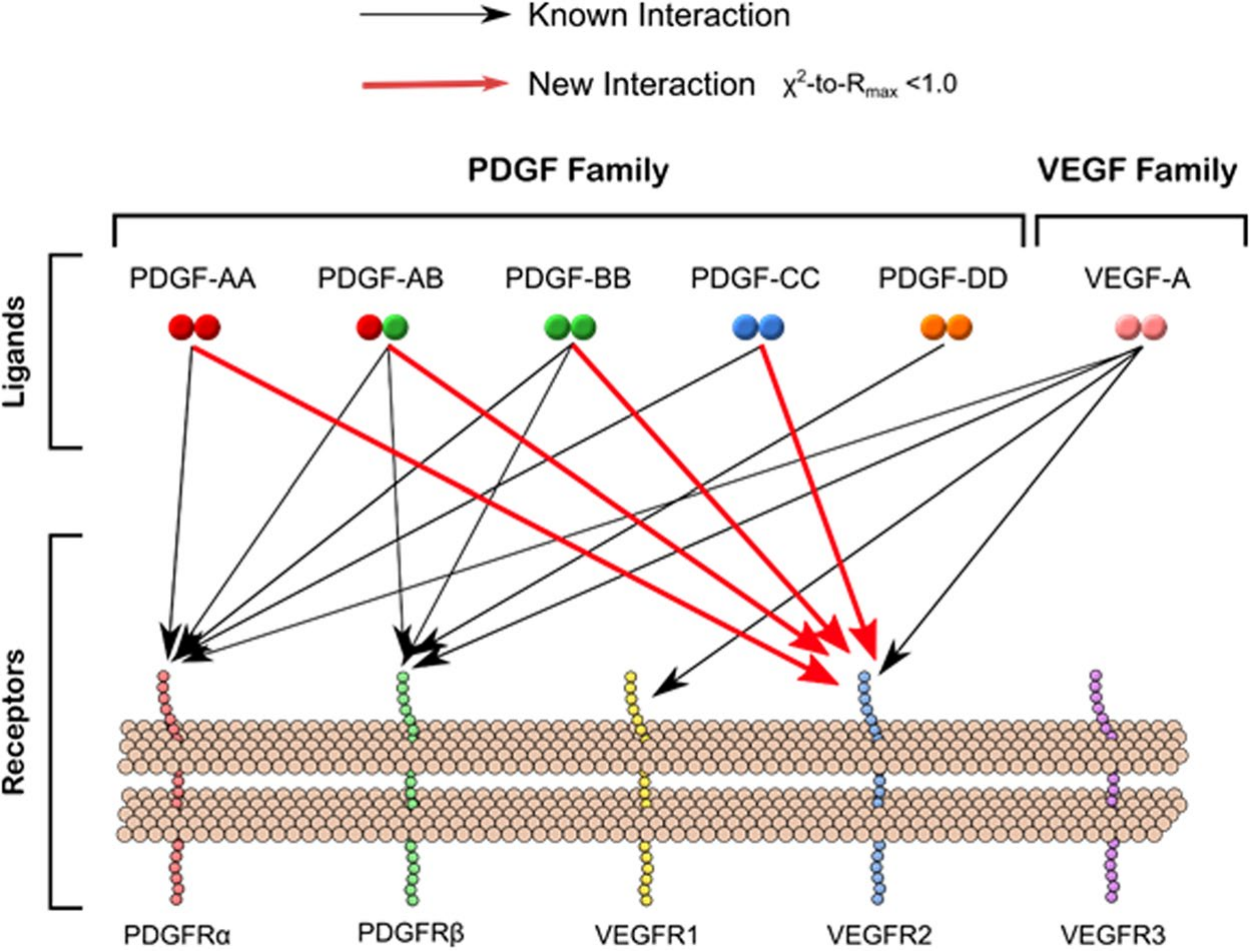
Summary of novel cross-family VEGF and PDGF ligand-receptor interactions. Schematic illustrates an updated view of the VEGF and PDGF ligand-receptor binding patterns, adding newly discovered PDGF-VEGFR interactions. Specifically, only new interactions where the kinetic analysis fit χ^2^-to-R_max_ ratio < 1.0 were included. Previously-known interactions are indicated with grey lines, and newly-found interactions are indicated with red lines.

We identified novel interactions between PDGF-AA, -AB, -BB, and -CC with VEGFR2 (Fig. 6A–D, respectively). These interactions all exhibited classic signal responses, characterized by a steady signal increase upon ligand injection, association-phase, followed by a steady signal decrease during buffer injection, dissociation-phase (Fig. 6). We show examples of this in PDGF:PDGFR binding (Fig. 5), and all were all found to have χ^2^-to-R_max_ < 1.0, indicating true 1:1 Langmuir interactions (Supplementary Table S1.) There was no evidence of PDGF interactions with VEGFR1 or VEGFR3 (Supplementary Fig. S1). A summary of the novel interactions identified can be found in Fig. 7. When we quantified kinetic binding rates (k_a_ and k_d_) and affinity constants (K_D_ = k_d_/k_a_), we observed that PDGF-CC:VEGFR2 had an affinity constant of the same order of magnitude as native VEGF-A:VEGFR2 binding with K_D_ (-CC:R2) ~70 pM versus (VEGF-A:R2) ~10 pM. The other PDGFs presented VEGFR2 binding affinities with decreasing strength as follows: PDGF-AB > -BB > -AA. Another notable finding was that PDGF-CC bound VEGFR2 at an affinity greater than or equal its binding to either PDGFR (Fig. 5A). PDGF-AA:VEGFR2 was a higher affinity, but similar order of magnitude as PDGF-AA:PDGFRα binding, K_D_ (-AA:R2) = 100 nM, vs. (-AA:Rα) = 660 nM. PDGF-AB:VEGFR2 binding was higher affinity than its binding to PDGF-AB:PDGFRs with K_D_ (-AB:R2) = 110 pM vs. (AB:PDGFRs) >800 nM. Additionally, PDGF-BB:VEGFR2 binding was higher affinity than its binding to PDGFRs, K_D_ (-BB:R2) = 37 nM vs. (-BB:PDGFRs) >400 nM (Fig. 5A). The exact values obtained for binding affinities can be found listed in Supplementary Table S2.

### Association and dissociation rate constants reveal affinity trends

The affinity of the PDGF:VEGFR2 is best understood by examining the association and dissociation rate constants. Here we observe that the high-VEGF:VEGFR2 affinity is attributed to fast association, ~10^6^ M^−1^ s^−1^ (Fig. 5B), and slow dissociation, ~1 day^−1^ (Fig. 5C.) Only the PDGF-CC:PDGFRβ association nears these VEGF-A:VEGFR2 association dynamics, which supports its high-affinity binding to PDGFRβ (Fig. 5A). All other association dynamics are <~10^5^ M^−1^ s^−1^. The slow VEGF:VEGFR2 dissociation rate constant is exceeded by PDGF-AB:VEGFR2, ~1/7 days (i.e. dissociating on average once per week), explaining its ~100 pM binding affinity (Fig. 5A). All other dissociation rate constants are “faster”—i.e. higher values—in the range 1/minutes and 1/hours (i.e. one molecule dissociates on average per minute and per hour, approximately). (The exact values obtained for association and dissociation rate constants can be found listed in Supplementary Table S2.)

### Model construction and validation

**Figure 8:**
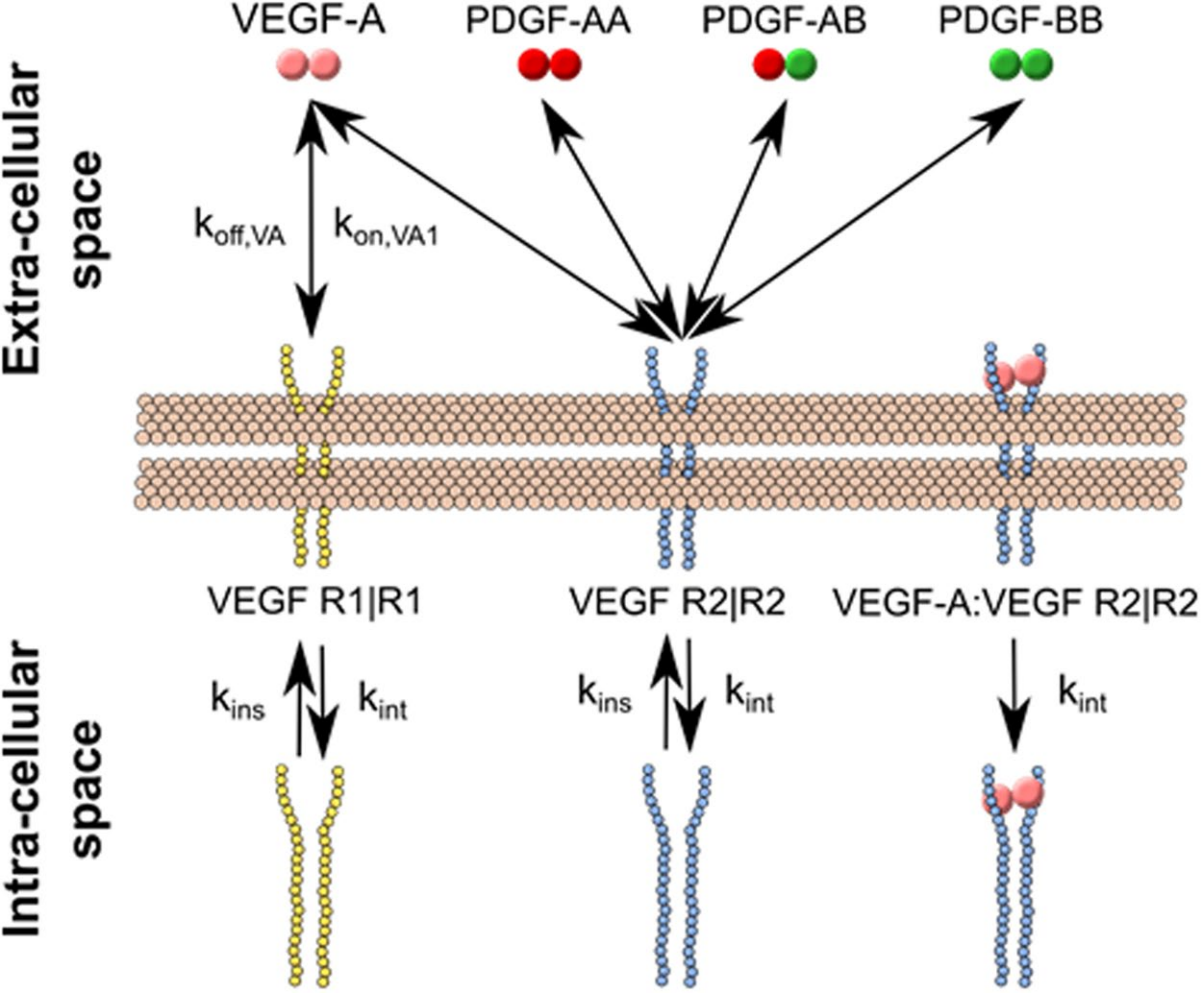
Qualitative model of endothelial cell VEGF and PDGF ligand-receptor binding. Schematic illustrates basic structure of computational model of VEGF-A:VEGFR and PDGF:VEGFR2 signaling for endothelial cells. Non-ligated dimers of VEGFR1 and VEGFR2 are inserted and internalized at a constant rate (k_ins_ and k_int_, respectively). Ligand-receptor binding for VEGFs and PDGFs as found in this study are incorporated. Ligated-receptor complexes are internalized at a constant rate k_int_.

**Figure 9:**
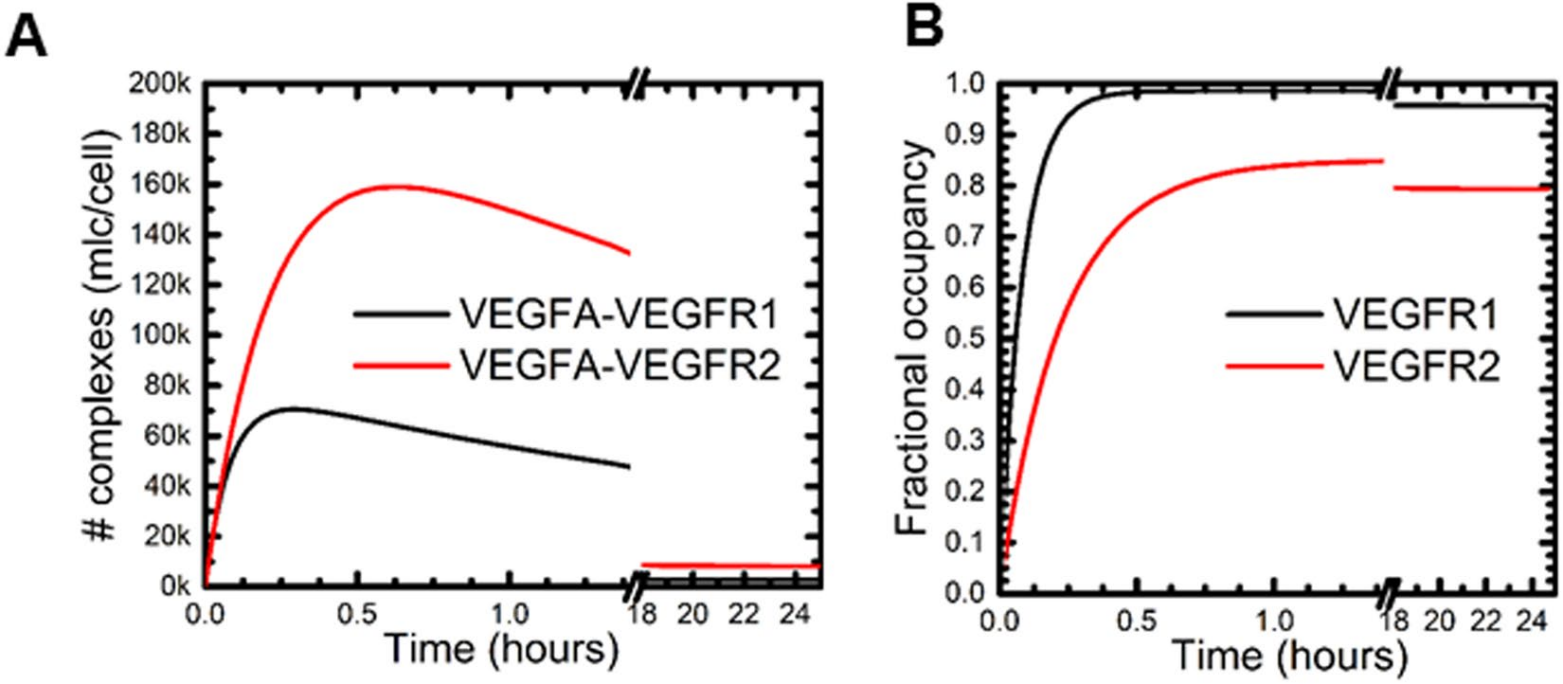
Recapitulation of VEGF-A:VEGFR benchmark model. All rate parameters and species concentrations taken from original study. **(A)** Number of VEGFA-receptor complexes formed over time) and **(B)** Fractional receptor occupancy for VEGFR1 and VEGFR2 over time, defined as: (# VEGFR1 complexes)/(total # VEGFR1) and (# VEGFR2 complexes)/(total # VEGFR2) respectively.

To predict the significance of PDGF-VEGFR2 cross-family binding, we derived the ordinary differential equations describing these ligand-receptor interactions based on the law of mass action. Klipp *et al*. and Linderman & Lauffenburger offer comprehensive explanation of this approach for representing protein-protein interactions^46–50^. Towards this goal, we simulated two sets of models governing VEGFR ligation: one describing the canonical, uni-family VEGF-A:VEGFR binding and one describing cross-family binding that includes both the canonical uni-family VEGF:VEGFR and our newly derived cross-family PDGF:VEGFR2 binding (PDGF-AA, -AB, & -BB with VEGFR2) (see model schematic Fig. 8). We do not include PDGF-CC in the model because PDGF-CC has not been found to be expressed in humans under healthy or cancer conditions, but is instead observed following a an ischemic event like a stroke or myocardial infarction^51^. We recapitulated the benchmark, uni-family VEGF-A:VEGFR model by Mac Gabhann^52^, showing consistent VEGFR1 and VEGFR2 fractional occupancies (Fig. 9A) and maximal responses (Fig. 9B).

We analyzed these two models for ligand concentrations found in healthy physiology, breast cancer, and anti-VEGF-A therapy in breast cancer. Bevacizumab is used as the basis of the anti-VEGF-A treatment, implemented as a binding interaction between bevacizumab and non-receptor-bound VEGF-A. Model equations and parameters are listed in full in Supplementary Files 1 and Supplementary Table S4, respectively.

### Localized PDGF cross-family binding enhances VEGFR2 activation in health and cancer

**Figure 10:**
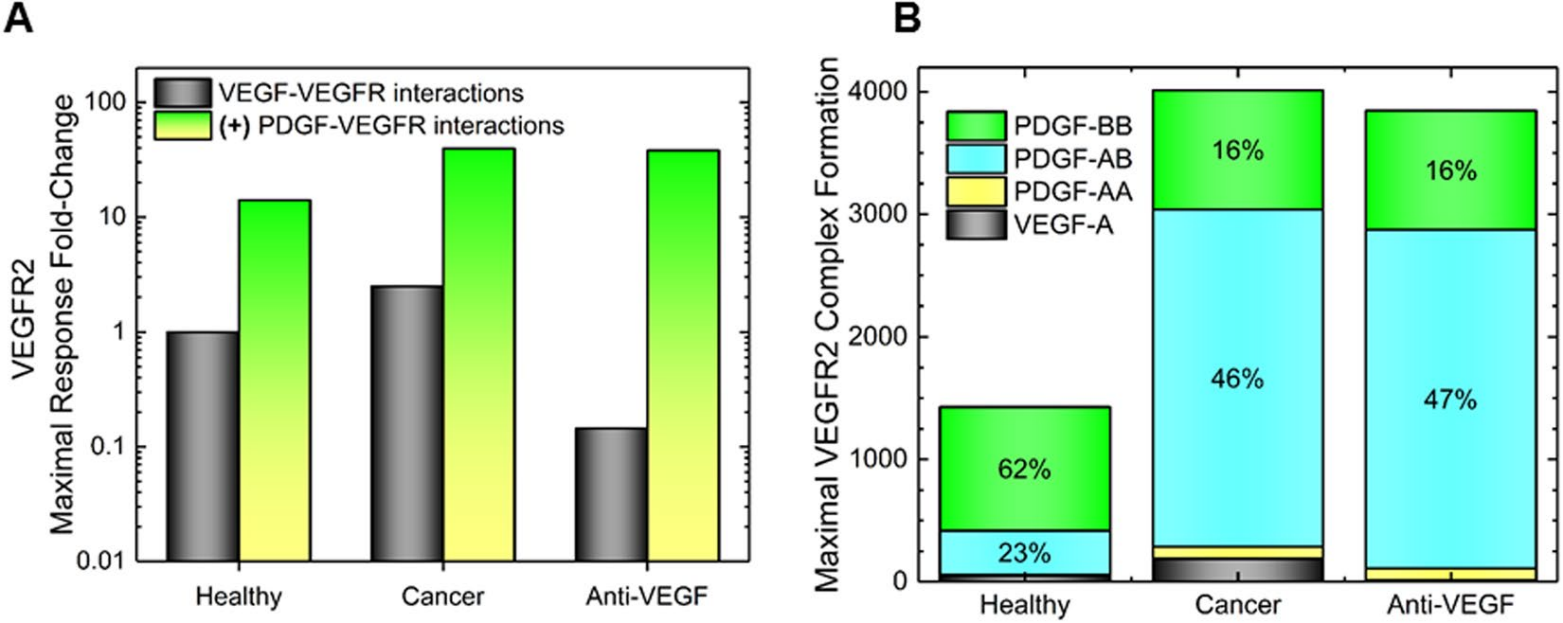
VEGF and PDGF ligation of VEGFR2 ligation in healthy, cancer, and anti-VEGF treated cancer conditions. (**A**) Fold-change in maximal ligand-VEGFR2 binding in different physiological conditions with and without PDGF-VEGFR interactions. Physiological conditions use VEGFA, PDGF-AA, -AB, -BB, VEGFR1 and VEGFR levels found in serum and healthy endothelial cells. Cancer conditions use serum levels and tumor endothelial cell levels found in breast cancer. Anti-VEGF treated cells use cancer conditions in addition to the introduction of bevacizumab, a monoclonal antibody targeting VEGF-A. **(B)** Ligand proportional composition in VEGF and PDGF cross-family model.

We predict increased VEGFR2 complex formation in every case where PDGF:VEGFR2 cross-family interactions are considered. First, the cross-family model in comparison to the uni-family model predicts a 14-fold increase in VEGFR2 ligation under healthy physiology (Fig. 10A). Similarly, the cross-family model predicts a 15-fold increase in VEGFR2 ligation in breast cancer xenograft-derived endothelial cells over the canonical, uni-family framework (Fig. 10A). Also, we observe that an anti-VEGF drug, like bevacizumab, would only contribute a ~5% decrease in VEGFR2 ligation when cross-family binding is considered in breast cancer xenograft-derived endothelial cells (Fig. 10A). Therefore, we predict that cross-family interactions may allow VEGFR2 complex formation to remain relatively unchanged when bevacizumab is administered, despite the fact that PDGFs bind VEGFR2 with affinities ~2 orders of magnitude weaker than VEGF-A (Fig. 10A).

**Figure 11:**
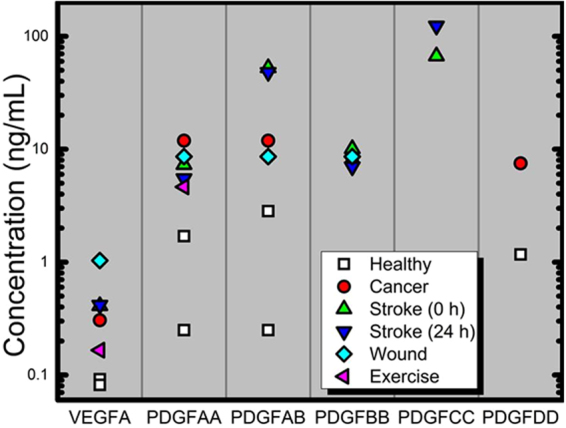
Meta-analysis of healthy and pathological VEGF and PDGF serum concentrations. A summary of known serum values for VEGF-A, PDGF-AA, -AB, -BB, -CC and –DD under healthy, breast cancer, and post-stroke (both at 0 hr and following 24 hr) conditions, wound fluid, and post-exercise. See Supplementary Table S5 for a complete summary of measured serum concentration values with references to the original studies.

The increased VEGFR2 ligation that we predict under a cross-family paradigm is attributed to the 10–100-fold higher PDGF to VEGF concentrations in serum under healthy physiological conditions. Indeed, we have included a meta-analysis comparing serum concentrations of VEGF-A and PDGFs across healthy and pathological conditions to contextualize these differences (Fig. 11). Such high PDGF concentrations may subsequently enable PDGFs to account for up to ~96% of VEGFR2 ligation under healthy conditions (Fig. 10B). Similarly, breast cancer patient serum is measured to have 25-to-40-fold higher PDGF levels than VEGF (Fig. 10A), so when considering VEGF-A and PDGFs, we predict up to 90% steady state ligation by PDGFs in breast cancer and nearly 100% ligation by PDGFs under conditions of anti-VEGF therapy (Fig. 10B). Our model suggests that this buffering of angiogenic signaling during anti-VEGF therapy may be mediated as follows: PDGF-AB >PDGF-AA >PDGF-BB >VEGF-A (Fig. 10B).

As previously described, we do not include PDGF-CC in the model given its limited expression pattern (e.g., ischemic event)^51^. However, we expect that PDGF-CC:VEGFR2 will dominate VEGFR2 ligation following ischemic damage for two reasons: (1) after ischemic injury PDGF-CC has a 150–300 fold greater serum concentration than VEGF-A (Fig. 11), and (2) PDGF-CC has similar, high-affinity (~100 pM) VEGFR2 binding as canonical VEGF-A:VEGFR2. Furthermore, we predict that PDGF-CC:VEGFR2 ligation would dominate whenever PDGF-CC serum concentrations are 2-fold greater than VEGF-A. We can extend a similar analysis to the other PDGF ligands. Our meta-analysis of PDGF and VEGF serum concentrations would also suggest that in every case where any PDGF serum concentration is at least 25-fold greater than VEGF, then PDGF:VEGFR2 ligation should significantly modulate VEGFR2 signaling. This would include in cancer, post-stroke, and during exercise (Fig. 11).

## Discussion

In this study, we established a χ^2^-to-R_max_ cut-off (where values are < 1.0) for distinguishing non-binding and binding interactions, which we used in the discovery of new PDGF-VEGFR interactions: (1) PDGF-AA to VEGFR2, (2) PDGF-AB to VEGFR2, (3) PDGF-BB to VEGFR2, and (4) PDGF-CC to VEGFR2 (summarized in Fig. 7). We measured both the kinetics of these new interactions; and for the first time, we measured the kinetics for VEGF-A:PDGFRβ^27^ binding. In a cross-family signaling system, where several ligands compete to bind with the same receptor, the ligand concentrations and ligand-receptor binding kinetics determine which ligand(s) dominate signaling. Therefore, our computational analysis provided quantitative evidence that PDGF:VEGFR2, cross-family binding should significantly participate in VEGFR2 ligation under conditions of health physiology, breast cancer, and pathologies where PDGFs significantly outnumber VEGFs. Furthermore, our modeling offers predictions on how PDGF:VEGFR2 interactions may sustain VEGFR2 ligation when VEGF-A is inhibited (e.g., bevacizumab, ziv-aflibercept). Together these findings offer new insight into several fields of research, including anti-angiogenic drug development and cancer, which we contextualize herein.

### Cross-family interactions may shed new light on anti-angiogenic drug development

Our discovery of cross-family interactions can advance anti-angiogenic drug development, which is currently guided by a uni-family view^53,54^. Anti-VEGF success has been limited, due to both intrinsic resistance and acquired resistance (relapse)^55^. Several mechanisms have been proposed to explain anti-VEGF resistance, a few include: (1) Increased mural cells: some tumors resist anti-angiogenic drug treatment via increased pericyte coverage of tumor vessels^55,56^, providing a permeability barrier to anti-VEGF therapy. (2) Increased tumor invasiveness: tumor undergoes enhanced metastasis into healthy tissue as a means of co-opting existing vasculature^57^; thereby seeking to avoid hypoxia or nutrient deprivation and bypassing anti-VEGF therapy. (3) Ancillary growth factors: some tumors secrete additional growth factors, such as PDGFs and fibroblast growth factors^55^, which enable ancillary angiogenic signaling axes to bypass anti-VEGF therapy. The latter mechanism may be supported by our findings if PDGF:VEGFR2 complexes lead to VEGFR2 activation and angiogenic signaling. Therefore, future work should determine how PDGF:VEGFR2 ligation regulates native VEGFR2 signaling.

Cross-family signaling may also aid our understanding of multi-target small molecule inhibitors, like sorafenib and sunitinib. Sorafenib inhibits VEGFR2, VEGFR3 and PDGFRβ^33^, while sunitinib inhibits PDGFRβ and all VEGFRs^58^. When viewed through the uni-family signaling lens, these small molecules would only inhibit uni-family interactions, i.e. blocking PDGFR autophosphorylation would render PDGF ligands as non-functional. This perspective does not explain why patients also relapse on these cross-family anti-angiogenic drugs. Under a cross-family paradigm, blocking VEGFR and PDGFR would shift VEGF and PDGF binding to receptors of other signaling families. Some possibilities could be other, structurally similar tyrosine kinase receptors with angiogenic function, such as: nerve, fibroblast, and transforming growth factor receptors^59–61^. Our work suggests that drug resistance to multi-target therapeutics can be better informed and overcome by identifying the extent of cross-family binding.

### The tip of the iceberg? Structural analysis can guide further cross-family discovery

Our results open the possibility that that other cross-family interactions exist, but remain undiscovered. Experimentally screening for interactions between every known cytokine with every known receptor would be cost- and time-prohibitive. Instead, structural analysis of shared ligand and receptor structural motifs can guide further discovery. For example, VEGFRs and PDGFRs are tyrosine kinase receptors: PDGFRs have 5 extracellular immunoglobulin-like (IgG-like) domains^62^ and VEGFRs have 7 extracellular IgG-like domains in VEGFRs^63^. Of these, domains 2–3 are responsible for high-affinity binding of VEGF-A:VEGFR1^64^ and VEGF-A:VEGFR2^65^. Domains 2–3 are highly-conserved within PDGFRs, where they play an identical role of housing the PDGF binding site. The common ligand-binding domains suggests that PDGFs could utilize these sites to bind VEGFRs. Moreover, VEGF and PDGF are similar in quaternary structure: VEGF and PDGF have only a 1.93 Å root mean square difference between overlapping structures^66^ (Fig. 1B). VEGFs and PDGFs also share structural motifs in their receptor-binding regions^63^. In fact, PDGF ligands have been superimposed within the VEGF-A:VEGFR2 binding domains, illuminating the possible confirmations for the interactions we report^29^. Additionally, VEGFRs and PDGFRs have a similar intracellular structure: having an intracellular split tyrosine-kinase domain^67,68^. Such a feature may lend the possibility of agonist or partial agonist action via cross-family binding.

Cysteine knot ligands^66,69^ with some of these VEGF-PDGF structural properties should also be investigated; these include: nerve growth factors, fibroblast growth factors, transforming growth factors, and even glycoprotein hormones, due to their structural similarity to PDGF^66^. Examining other receptors that exhibit multi-ligand binding would offer a good template for understanding the possibilities in cross-family binding. For example, early work by Pennock *et al*. explored whether VEGF-A served as a cross-family agonist or antagonist for the PDGF receptors, finding the ligand acted antagonistically^70^. Outside the VEGFs and PDGFs, the nicotinic acetylcholine receptor is well known for having several possibilities for receptor assembly, and several molecules, other than nicotine that can bind with agonist-partial agonist-antagonist affect (e.g., epibatidine, choline, etc.)^71^. The transforming growth factor-β superfamily also offers a good template to examine multi-binding interactions, where extensive cross-family ligand:receptor binding between the subgroups, transforming growth factor-βs, bone morphogenetic proteins (BMPs), growth and differentiation factors (GDFs) and the activin/inhibins^72^ are responsible for maintaining a complicated regulatory network^73^. Structural analysis, therefore, can enable a focused screening of likely cross-family binding partners.

### Computational modeling will drive conceptual understanding of cross-family signaling

Computational modeling has provided new insight into the uni-family activation of several growth factor-receptor families, including EGF^74^, FGF^75^, PDGF^76^, and VEGF^3,50,77–81^. Here, our PDGF—VEGFR2 modeling and analysis predicted conditions where PDGF—VEGFR2 binding prevails, which included normal physiological conditions. While we do find the high-affinity of these cross-family interactions surprising, we do not find the modeling results to be surprising. It follows that when incorporating a new population of ligands—at concentrations higher than VEGF-A, and which bind the same receptor at affinities either slightly lower or similar to VEGF-A—the receptor would be substantially ligated by these new ligands. We expect that these patterns of ligated-receptor dominance could change when different healthy biological conditions are considered, such as when receptor trafficking rates are altered by changes in blood flowrate-induced sheer stresses^82^. As we continue exploring this new paradigm, additional models will need to determine how cross-family receptor occupancy translates to various receptor activation landscapes. An elegant computational framework for examining the possible receptor landscapes is the recent work on how bone morphogenetic proteins (BMP) and their receptors achieve multiple activation profiles^83^. We expect that further computational modeling of cross-family signaling will enable a similar understanding of receptor activation landscapes (full-agonist, partial-agonist, partial antagonist, and full-antagonist), receptor activation dynamics (fast/burst, slow/sustained, etc.), and effector efficiencies.

Another computational approach that should be enlisted for understanding maximal canonical signaling is the recent “meta-model” from our laboratory, which modeled receptor activation across eight, canonical ligand-receptor pairs^84^. This meta-model offered ranked predictions of receptor signaling, based on the integrated receptor activation response: PDGFRβ >IGFR1 >EGFR >PDGFRα >VEGFR1 >VEGFR2 >Tie2 >FGFR1. Because these receptors activate similar second messengers, this ranking of canonical (uni-axis) signaling provided the first guide for manipulating signaling in cells carrying combinations of these receptors. We envision that a multi-family model that incorporates the foundational model we present here, signaling landscapes^83^, and growth factor receptor hierarchy^84^ will offer novel predictions for maximally tuning signaling. Furthermore, we will test more conditions than are accessible by experiment to understand the multiple cross-family signaling permutations.

### Future studies: Filtering novel interactions via the χ^2^-to-R_max_ heuristic

Once structural analysis predicts additional cross-family partners, validation can use a similar SPR approach, as we established here. Towards such goals, researchers may apply the χ^2^-to-R_max_ ratio that we discuss to designate “true” interactions from non-specific, non-1:1 Langmuir interactions. Indeed, SPR can often produce large response curves that suggest binding, even where no interaction exists. Such curves are primarily due to two types of non-specific binding: (1) sensor-ligand interactions^85^ and (2) receptor-ligand interactions^86^. The former can be corrected by coating a flow cell with a non-binding reference protein. Without the use of a reference protein, non-specific effects would artificially inflate the observed binding affinity strength (lower K_D_). For this study we used angiopoietin-4 as a reference (see Materials and Methods); thus minimizing non-specific interactions between ligand and chip. However, previous SPR measurements of PDGF:PDGFR interactions, did not use a reference protein^45^. We believe this critical difference explains our different affinities, with prior studies measuring a weaker binding affinity. Non-specific ligand-receptor interactions can also be accounted for when you consider that such interactions would not likely follow a 1:1 Langmuir binding model. This is due to the fact that many non-specific interactions are mediated by transient electrostatic attractions between amino acid residues on the ligand and receptor. These transiently-interacting residues can be distributed throughout both proteins—are therefore not site-specific—and should not follow a 1:1 interaction pattern^86–88^ indicated by the χ^2^-to-R_max_ ratio. Future measurements of cross-family binding should apply our finding that χ^2^-to-R_max_ ratio >1.0 is attributed to non-specific binding.

## Conclusion

These studies offer new interpretations of tyrosine kinase receptor signaling: a cross-family paradigm. We provide standards for kinetic analyses of novel interactions with broad implications for tyrosine kinase receptor signaling. Through computational modeling, we further predict that cross-family interactions may significantly alter signaling. From these results, we propose a new mechanism for anti-angiogenic drug resistance, which may aid drug development.

In addition to the applications of this work for tumor angiogenesis, PDGF-VEGF cross-family signaling should affect our understanding of cardiovascular-dependent diseases, governed by angiogenic signaling, including: wound healing^13^, exercise^89^, and other cardiovascular pathologies^6,90^. Moreover, it should enable improved vascularization in the areas of tissue engineering and regenerative medicine^89,91–93^.

Finally, our work suggests that structural similarities in signaling ligands and receptors could indicate cross-family binding patterns and that this phenomenon could be common across cell signaling (e.g., agonist/partial agonist paradigm). These and any new interactions can and should be illuminated through structural analysis and computational modeling.

## Materials and Methods

### Surface plasmon resonance (SPR)

All SPR studies were performed with the BIAcore 3000 instrument (Biacore International AB, Uppsala, Sweden) at 25 °C on dextran-coated gold sensor chips (CM5, Research grade, GE Healthcare Bio-sciences AB, Uppsala). The BIAcore 3000 divides CM5 sensor chips into four separate flow cells. We immobilized a different receptor protein in each flow cell: The first cell was reserved for measuring non-specific binding by immobilizing recombinant angiopoietin-4 (Cat. #964-AN-025/CF, R&D Systems) to a flow cell: it has no known interaction with VEGFs or PDGFs. Three different receptors were immobilized to the three remaining flow cells. *Running buffer*: 1x HBS-EP pH 7.4 (10 mM HEPES, 3 mM EDTA, 150 mM NaCl, 0.005% TWEEN-20, cat. # BR100188, GE Life Sciences).

### Optimizing immobilization conditions

Human recombinant VEGFR1 (Cat. #321-FL-050/CF, R&D Systems), VEGFR2 (Cat. #357-KD-050/CF, R&D Systems), VEGFR3 (Cat. #349-F4–050, R&D Systems), PDGFRα (Cat #322-PR-050/CF, R&D Systems) and PDGFRβ (Cat. #385-PR-100/CF, R&D Systems) were immobilized on flow cells by first performing pre-concentration studies to determine optimal receptor-immobilization pH (Supplementary Table S6), as follows: 20 µg/mL receptor + 10 mM acetate buffers ranging from pH 3.5 to 0.5–1.0 below the protein isoelectric point (Supplementary Table S6) were prepared. Known receptor-ligand was introduced at 5 µL/min (association), followed by 5-µL injection of ethanolamine-HCL (GE Healthcare AB, Uppsala, Sweden) to clear the surface. We selected the optimal acetate buffer pH for each protein based on (1) the maximum level of protein immobilization reached and (2) the rate of immobilization observed in the pre-concentration study sensograms.

### Protein immobilization

Receptor proteins were immobilized to the sensor dextran matrix via amine coupling. Flow cells were activated by injecting 35 μL of a 1:1 volumetric mixture of 0.05 M NHS (N-hydroxysuccinimide) and 0.2 M EDC (1-ethyl-3-(3-dimethylaminopropyl) carbodiimide hydrochloride) at 5 μL/min. 20 µg/mL of the aforementioned recombinant receptor in 10 mM acetate buffer at its optimal pH was injected at 5 µL/min until the target level was reached (approximately 200–500 R.U. of immobilized receptor). After sufficient protein coupling (~200–500 R.U. immobilized protein), the surface was de-activated by 35 μL ethanolamine (ethanolamine hydrochloride-NaOH pH 8.5, GE Healthcare AB) (Supplementary Fig. S2).

### Ligand-receptor association and dissociation kinetics

Fresh ligand solutions were prepared at 40, 20 and 10 nM in HBS-EP running buffer each experimental day, including: human recombinant VEGFA (R&D Systems, Cat. #293-VE-010), PDGF-AA (Cat. #221-AA-050), -AB (Cat. #222-AB-050), -BB (Cat. #220-BB-050), -CC (Cat. #1687-CC-025) and -DD (Cat. #1159-SB-025) (Note: All ligands were obtained from R&D Systems, and were human recombinant proteins). 120 μL of each ligand solution was injected into flow cells containing immobilized receptor and Ang-4—a reference for non-specific binding—at 30 μL/min (association). This was followed by a 10 min running-buffer injection (dissociation). Between each sample, we injected, in series, 5 μL of 5 mM HCl and 5 μL of 10 mM NaOH at 5 μL/min to remove any remaining bound ligand. We repeated this cycle for each concentration tested (40, 20, and 10 nM, summarized in Fig. 2). Each concentration series were performed in triplicate.

### Kinetic analysis

The raw ligand:receptor sensograms were aligned and the background, non-specific binding was subtracted using the sensogram trace from the ligand:Ang-4 flow cell (Supplementary Figure S3). Both the raw ligand:receptor and ligand:Ang-4 sensogram curves were obtained within the BIAcore 3000’s detection window (10–70,000 R.U.) for all interactions^94^ to ensure detected interactions did not represent system noise. Post-subtraction sensograms with a negative-sloped association phase were excluded from global kinetic fitting and not considered interactions. BIAevaluation removes momentary signal spikes resulting from transient air bubbles.

Global analysis is considered to produce more accurate results than fitting of a single response curve, so global fitting was performed with BIAevaluation software (Version 4.1.1, GE Healthcare), which follows 1:1 Langmuir adsorption isotherm (Equation 1)^95^. The software applies nonlinear least squares analysis to determine association (*k*_*a*_) and dissociation (*k*_*d*_) rates fitting best to multiple response curves simultaneously. Additionally, the software provides the goodness-of-fit parameter χ^2^ and the peak magnitude of the signal response, R_max_.

1 $$R+L\mathop{\leftrightarrow }\limits^{{k}_{a},{k}_{d}}RL$$

### Classifying binding

Both the instrument manufacturer (BIAcore) and previous researchers have suggested that when fitting kinetic rate constants using global analysis, a χ^2^-to-R_max_ value (a measure of noise-to-signal) < 0.2 is ideal for confidence in the kinetic parameters obtained when studying known interactions^35,36,96^. A low noise-to-signal indicates that the sensogram signal includes minimal contributions from the following three noise-factors: (1) overall instrument noise, (2) heterogeneities in immobilized receptor or ligand, and (3) non-specific interactions. Using ligand:receptor pairs with known interactions and pairs known not to interact, we established a cut-off value of χ^2^-to-R_max_ < 1.0 that differentiates real, 1:1 Langmuir interactions, from predominantly non-specific interactions, where χ^2^-to-R_max_ > 1.0. We arrived at this via experimentally fitting known interactions and non-interactions (Supplementary Table S1). We then applied the χ^2^-to-R_max_ < 1.0 heuristic to classifying the interactions observed in PDGF:VEGFR binding.

### Single-cell model of membrane receptor activation

We recapitulated the uni-family (VEGF:VEGFR) single cell model described by Mac Gabhann *et al*., consisting of 7 ordinary differential equations (ODEs) derived using the law of mass action. The cell is represented as a single compartment^52^ that represents both the extracellular space and plasma membrane. We extended this to a dual-family model, including the 7 ODEs describing VEGF:VEGFR ligation plus an additional 8 ODEs describing PDGF:VEGFR2 signaling. The model equations and parameters are implemented in MATLAB using the SimBiology toolbox. Steady-state and dynamic solutions are computed using the ode15 solver for 24 simulated hours. A complete description of model equations for both uni-family and dual-family models are provided in Supplementary Text 1.

### Model parameters

Parameters are based upon 7 guidelines. (1) VEGFR1 and VEGFR2 are inserted into the plasma membranes at fixed rates^52^. (2) Ligand-free receptors and ligand-bound receptors are internalized from the plasma membrane at fixed rates^97^ (see Supplementary Table S4 for full summary of kinetic parameters). (3) VEGFR dimerization is not modeled explicitly, reflecting recent work that suggests VEGF receptors are initially pre-dimerized, and ligand-binding initiates a conformational change that enables phosphorylation and down-stream signaling events^98^. (4) Ligand secretion and ligand clearance are not modeled to better represent a localized cell environment without the influence of the systemic dynamics^52^. (5) Ligand concentrations are derived from known serum concentrations (Supplementary Table S5). (6) Benchmark kinetic and concentration parameters are used only to recapitulate the benchmark. (7) The cross-family model applied updated kinetic parameters via SPR studies herein, (Supplementary Table S2) and updated VEGFR concentrations, which were based on recent quantitative flow (qFlow) cytometry measurements^99–102^. All model parameters, their descriptions, and their values are provided in Supplementary Table S4.

We modeled anti-angiogenic drug treatment by incorporating the anti-VEGF-A antibody, bevacizumab. In the model, bevacizumab reversibly binds free VEGF-A, with kinetic constants previously-determined via SPR^103^. Drug treatment was modeled as a single, initial bevacizumab dose. The dose concentration used was adopted from an earlier VEGF computational model that based the dosage on patient plasma concentrations following the administration of bevacizumab in advanced cancer treatment^104,105^. Thus, this value correlates with the systemic level of bevacizumab. We excluded bevacizumab clearance and ‘secretion’ (the movement of bevacizumab from plasma into the area around endothelial cells), to examine the concentrations individual endothelial cells would experience.

### Data availability Statement

The datasets generated during and/or analyzed during the current study are available from the corresponding author on reasonable request.

## Electronic supplementary material

Supplementary File

## References

[1] Simons M, Gordon E, Claesson-Welsh L (2016). Mechanisms and regulation of endothelial VEGF receptor signalling. Nat Rev Mol Cell Biol.

[2] Chang CC (2010). Angiogenesis in a microvascular construct for transplantation depends on the method of chamber circulation. Tissue engineering. Part A.

[3] Mac Gabhann F, Qutub AA, Annex BH, Popel AS (2010). Systems biology of pro-angiogenic therapies targeting the VEGF system. Wiley Interdisciplinary Reviews: Systems Biology and Medicine.

[4] Mac Gabhann F, Annex BH, Popel AS (2010). Gene therapy from the perspective of systems biology. Current opinion in molecular therapeutics.

[5] Gupta R, Tongers JJ, Losordo DW (2009). Human studies of angiogenic gene therapy. Circulation Research.

[6] Annex BH (2013). Therapeutic angiogenesis for critical limb ischaemia. Nat Rev Cardiol.

[7] Sedighiani F, Nikol S (2011). Gene therapy in vascular disease. Surgeon.

[8] Bergers G, Benjamin LE (2003). Tumorigenesis and the angiogenic switch. Nature Reviews. Cancer.

[9] Erber R (2004). Combined inhibition of VEGF and PDGF signaling enforces tumor vessel regression by interfering with pericyte-mediated endothelial cell survival mechanisms. FASEB journal: official publication of the Federation of American Societies for Experimental Biology.

[10] Casanovas O, Hicklin DJ, Bergers G, Hanahan D (2005). Drug resistance by evasion of antiangiogenic targeting of VEGF signaling in late-stage pancreatic islet tumors. Cancer Cell.

[11] Attanasio S, Snell J (2009). Therapeutic angiogenesis in the management of critical limb ischemia: current concepts and review. Cardiology in review.

[12] Hsu CW (2015). Improved angiogenesis in response to localized delivery of macrophage-recruiting molecules. PLoS ONE.

[13] Shah, N. J. *et al*. Adaptive growth factor delivery from a polyelectrolyte coating promotes synergistic bone tissue repair and reconstruction. *Proceedings of the National Academy of Sciences of the United States of America* 1–6, 10.1073/pnas.1408035111 (2014). 10.1073/pnas.1408035111 PMC4156697 25136093

[14] Finley SD, Popel AS (2012). Predicting the effects of anti-angiogenic agents targeting specific VEGF isoforms. The AAPS journal.

[15] Koch S, Tugues S, Li X, Gualandi L, Claesson-Welsh L (2011). Signal transduction by vascular endothelial growth factor receptors. Biochem J.

[16] Ladomery MR, Harper SJ, Bates DO (2007). Alternative splicing in angiogenesis: The vascular endothelial growth factor paradigm. Cancer Letters.

[17] Biselli-Chicote PM, Oliveira ARCP, Pavarino EC, Goloni-Bertollo EM (2012). VEGF gene alternative splicing: pro- and anti-angiogenic isoforms in cancer. Journal of Cancer Research and Clinical Oncology.

[18] Bates DO (2002). VEGF165b, an inhibitory splice variant of vascular endothelial growth factor, is down-regulated in renal cell carcinoma. Cancer research.

[19] Konopatskaya O, Churchill AJ, Harper SJ, Bates DO, Gardiner TA (2006). VEGF165b, an endogenous C-terminal splice variant of VEGF, inhibits retinal neovascularization in mice. Molecular vision.

[20] Chappell JC (2016). Flt-1 (VEGFR-1) coordinates discrete stages of blood vessel formation. Cardiovascular Research.

[21] Carmeliet P, Collen D (1998). Vascular development and disorders: Molecular analysis and pathogenic insights. Kidney Int.

[22] Betsholtz C, Karlsson L, Lindahl P (2001). Developmental roles of platelet-derived growth factors. BioEssays.

[23] Battegay EJ, Rupp J, Iruela-Arispe L, Sage EH, Pech M (1994). PDGF-BB modulates endothelial proliferation and angiogenesis *in vitro* via PDGF beta-receptors. The Journal of cell biology.

[24] Brown DM, Hong SP, Farrell CL, Pierce GF, Khouri RK (1995). Platelet-derived growth factor BB induces functional vascular anastomoses *in vivo*. Proceedings of the National Academy of Sciences.

[25] Lindner V, Reidy MA (1995). Platelet-derived growth factor ligand and receptor expression by large vessel endothelium *in vivo*. The American journal of pathology.

[26] Martins RN, Chleboun JO, Sellers P, Sleigh M, Muir J (1994). The Role of PDGF-BB on the Development of the Collateral Circulation after Acute Arterial Occlusion. Growth Factors.

[27] Ball SG, Shuttleworth CA, Kielty CM (2007). Vascular endothelial growth factor can signal through platelet-derived growth factor receptors. The Journal of Cell Biology.

[28] Reigstad LJ (2003). Platelet-derived growth factor (PDGF)-C, a PDGF family member with a vascular endothelial growth factor-like structure. The Journal of biological chemistry.

[29] Muller YA, Christinger HW, Keyt BA, de Vos AM (1997). The crystal structure of vascular endothelial growth factor (VEGF) refined to 1.93 A resolution: multiple copy flexibility and receptor binding. Structure (London, England: 1993).

[30] Greenberg JI (2008). A role for VEGF as a negative regulator of pericyte function and vessel maturation. Nature.

[31] Greenberg JI, Cheresh DA (2009). VEGF as an inhibitor of tumor vessel maturation: implications for cancer therapy. Expert Opinion on Biological Therapy.

[32] Sava P, Cook IO, Mahal RS, Gonzalez AL (2015). Human microvascular pericyte basement membrane remodeling regulates neutrophil recruitment. Microcirculation.

[33] Majesky MW (2007). Developmental basis of vascular smooth muscle diversity. Arteriosclerosis, Thrombosis, and Vascular Biology.

[34] Karlsson R (1999). Affinity analysis of non-steady-state data obtained under mass transport limited conditions using BIAcore technology. Journal of molecular recognition: JMR.

[35] Murphy, M., Jason-Moller, L. & Bruno, J. Using Biacore to measure the binding kinetics of an antibody-antigen interaction. *Current protocols in protein science editorial board John E Coligan et al* Chapter 19, Unit 19.14–Unit19.14 (2006). 10.1002/0471142301.ps1914s45 18429303

[36] Biacore, A. B., July, E. & Biacore, A. B. *BIAevaluation Software Handbook*. *System* (Biacore AB, 1997).

[37] Nilsson I (2010). VEGF receptor 2/-3 heterodimers detected *in situ* by proximity ligation on angiogenic sprouts. EMBO J.

[38] Seifert RA (1989). Two different subunits associate to create isoform-specific platelet-derived growth factor receptors. Journal of Biological Chemistry.

[39] von Tiedemann B, Bilitewski U (2002). Characterization of the vascular endothelial growth factor-receptor interaction and determination of the recombinant protein by an optical receptor sensor. Biosensors and Bioelectronics.

[40] Cunningham SA, Tran TM, Arrate MP, Brock TA (2000). Characterization of Vascular Endothelial Cell Growth Factor Interactions with the Kinase Insert Domain-containing. The Journal of biological chemistry.

[41] Hart CE (1988). Two classes of PDGF receptor recognize different isoforms of PDGF. Science.

[42] Fleming TP, Matsui T, Aaronson SA (1992). Platelet-derived growth factor (PDGF) receptor activation in cell transformation and human malignancy. Experimental gerontology.

[43] Fretto LJLJ (1993). Mechanism of platelet-derived growth factor (PDGF) AA, AB, and BB binding to alpha and beta PDGF receptor. Journal of Biological Chemistry.

[44] Moriya, J. *et al*. Platelet-derived growth factor C promotes revascularization in ischemic limbs of diabetic mice. *Journal of Vascular Surgery* **59**, 1402–1409.e4 (2014). 10.1016/j.jvs.2013.04.053 23856609

[45] Lin X, Takahashi K, Liu Y, Derrien A, Zamora PO (2007). A synthetic, bioactive PDGF mimetic with binding to both alpha-PDGF and beta-PDGF receptors. Growth factors (Chur, Switzerland).

[46] Klipp, E., Liebermeister, W., Wierling, C., Kowald, A. & Herwig, R. *Systems Biology: A Textbook*. (Wiley-Blackwell, 2016).

[47] Horn F, Jackson R (1972). General mass action kinetics. Archive for Rational Mechanics and Analysis.

[48] Janes KA, Lauffenburger DA (2006). A biological approach to computational models of proteomic networks. Current Opinion in Chemical Biology.

[49] Lauffenburger, D. A & Linderman, J. J. Receptors: Models for binding, trafficking and signaling. **40**, (Oxford University Press, 1994).

[50] Weddell JC, Imoukhuede PI (2014). Quantitative Characterization of Cellular Membrane-Receptor Heterogeneity through Statistical and Computational Modeling. PLoS ONE.

[51] Rodríguez-González R (2013). Platelet derived growth factor-CC isoform is associated with hemorrhagic transformation in ischemic stroke patients treated with tissue plasminogen activator. Atherosclerosis.

[52] Mac Gabhann F (2004). Model of competitive binding of vascular endothelial growth factor and placental growth factor to VEGF receptors on endothelial cells. American journal of physiology. Heart and circulatory physiology.

[53] Albini A, Tosetti F, Li VW, Noonan DM, Li WW (2012). Cancer prevention by targeting angiogenesis. Nature Reviews Clinical Oncology.

[54] Meadows KN, Bryant P, Pumiglia K (2001). Vascular endothelial growth factor induction of the angiogenic phenotype requires Ras activation. The Journal of Biological Chemistry.

[55] Bergers G, Hanahan D (2008). Modes of resistance to anti-angiogenic therapy. Nat Rev Cancer.

[56] Jain RK, Booth MF (2003). What brings pericytes to tumor vessels?. Journal of Clinical Investigation.

[57] Rubenstein JL (2000). Anti-VEGF antibody treatment of glioblastoma prolongs survival but results in increased vascular cooption. Neoplasia (New York, NY).

[58] Christensen JG (2007). A preclinical review of sunitinib, a multitargeted receptor tyrosine kinase inhibitor with anti-angiogenic and antitumour activities. Annals of Oncology.

[59] Cantarella G (2002). Nerve growth factor-endothelial cell interaction leads to angiogenesis *in vitro* and *in vivo*. The FASEB journal: official publication of the Federation of American Societies for Experimental Biology.

[60] Lieu C, Heymach J, Overman M, Tran H, Kopetz S (2011). Beyond VEGF: Inhibition of the fibroblast growth factor pathway and antiangiogenesis. Clinical Cancer Research.

[61] Roberts AB (1986). Transforming growth factor type beta: rapid induction of fibrosis and angiogenesis *in vivo* and stimulation of collagen formation *in vitro*. Proceedings of the National Academy of Sciences.

[62] Kovalenko MVM, Kazlauskas A, William JL, Lane MD (2004). Platelet-Derived Growth Factor Receptor Family. Encyclopedia of Biological Chemistry.

[63] Starovasnik MA (1999). Solution structure of the VEGF-binding domain of Flt-1: comparison of its free and bound states. Journal of molecular biology.

[64] Wiesmann C (1997). Crystal structure at 1.7 A resolution of VEGF in complex with domain 2 of the Flt-1 receptor. Cell.

[65] Fuh G, Li B, Crowley C, Cunningham B, Wells JA (1998). Requirements for binding and signaling of the kinase domain receptor for vascular endothelial growth factor. Journal of Biological Chemistry.

[66] Muller YA (1997). Vascular endothelial growth factor: crystal structure and functional mapping of the kinase domain receptor binding site. Proceedings of the National Academy of Sciences of the United States of America.

[67] McTigue MA (1999). Crystal structure of the kinase domain of human vascular endothelial growth factor receptor 2: a key enzyme in angiogenesis. Structure (London, England: 1993).

[68] Chen PP-HH, Chen X, He X (2012). Platelet-derived growth factors and their receptors: Structural and functional perspectives. Biochimica et Biophysica Acta (BBA)-Proteins.

[69] Iyer S, Acharya KR (2011). Tying the knot: The cystine signature and molecular-recognition processes of the vascular endothelial growth factor family of angiogenic cytokines. FEBS Journal.

[70] Pennock S, Kazlauskas A (2012). Vascular endothelial growth factor A competitively inhibits platelet-derived growth factor (PDGF)-dependent activation of PDGF receptor and subsequent signaling events and cellular responses. Molecular and cellular biology.

[71] Drenan RM (2008). Subcellular Trafficking, Pentameric Assembly, and Subunit Stoichiometry of Neuronal Nicotinic Acetylcholine Receptors Containing Fluorescently Labeled a6 and b3 Subunits. Molecular pharmacology.

[72] Yadin D, Knaus P, Mueller TD (2016). Structural insights into BMP receptors: Specificity, activation and inhibition. Cytokine and Growth Factor Reviews.

[73] Nicklas D, Saiz L (2013). Computational modelling of Smad-mediated negative feedback and crosstalk in the TGF-β superfamily network. Journal of the Royal Society, Interface/the Royal Society.

[74] Wiley HS (2003). Computational modeling of the EGF-receptor system: a paradigm for systems biology. Trends in Cell Biology.

[75] Filion RJ, Popel AS (2004). A reaction-diffusion model of basic fibroblast growth factor interactions with cell surface receptors. Annals of Biomedical Engineering.

[76] Park CS, Schneider IC, Haugh JM (2003). Kinetic analysis of platelet-derived growth factor receptor/phosphoinositide 3-kinase/Akt signaling in fibroblasts. Journal of Biological Chemistry.

[77] Qutub AA (2009). A. S. Elongation, proliferation & migration differentiate endothelial cell phenotypes and determine capillary sprouting. BMC Syst Biol.

[78] Ji JW, Mac Gabhann F, Popel AS (2007). Skeletal muscle VEGF gradients in peripheral arterial disease: simulations of rest and exercise. American journal of physiology. Heart and circulatory physiology.

[79] Mac Gabhann F, Ji JW, Popel AS (2007). VEGF gradients, receptor activation, and sprout guidance in resting and exercising skeletal muscle. J Appl Physiol (1985).

[80] Vempati P, Mac Gabhann F, Popel AS (2010). Quantifying the Proteolytic Release of Extracellular Matrix-Sequestered VEGF with a Computational Model. PLoS ONE.

[81] Qutub A, Gabhann F, Karagiannis E, Vempati P, Popel A (2009). Multiscale models of angiogenesis. in. IEEE Engineering in Medicine and Biology Magazine.

[82] Chen K (1999). Mechanotransduction in Response to Shear Stress. The Journal of biological chemistry.

[83] Antebi, Y. E. *et al*. Combinatorial Signal Perception in the BMP Pathway. *Cell* **170**, 1184–1196 e24 (2017). 10.1016/j.cell.2017.08.015 PMC5612783 28886385

[84] Weddell, J. C. & Imoukhuede, P. I. Integrative meta-modeling identifies endocytic vesicles, late endosome and the nucleus as the cellular compartments primarily directing RTK signaling. *Integrative Biology* (2017). 10.1039/C7IB00011A 10.1039/c7ib00011a 28436498

[85] Fong CC, Wong MS, Fong WF, Yang M (2002). Effect of hydrogel matrix on binding kinetics of protein-protein interactions on sensor surface. Analytica Chimica Acta.

[86] Haseley SR, Talaga P, Kamerling JP, Vliegenthart JF (1999). Characterization of the carbohydrate binding specificity and kinetic parameters of lectins by using surface plasmon resonance. Analytical biochemistry.

[87] Drake AW (2012). Biacore surface matrix effects on the binding kinetics and affinity of an antigen/antibody complex. Analytical Biochemistry.

[88] Fivash M, Towler EM, Fisher RJ (1998). BIAcore for macromolecular interaction. Current Opinion in Biotechnology.

[89] Huntsman HD (2013). Mesenchymal stem cells contribute to vascular growth in skeletal muscle in response to eccentric exercise. American journal of physiology. Heart and circulatory physiology.

[90] Hirsch AT (2001). Peripheral Arterial Disease Detection, Awareness, and Treatment in Primary Care. JAMA: The Journal of the American Medical Association.

[91] Ebong EE, Spray DC, Tarbell JM (2011). The Glypican-1 HS Core Protein of the Glycocalyx is Important for Flow-induced Endothelial NOS Activation but not Cell Remodeling. The FASEB Journal.

[92] Oredein-McCoy O (2009). Novel factor-loaded polyphosphazene matrices: potential for driving angiogenesis. Journal of microencapsulation.

[93] Pence JC, Clancy KBH, Harley BAC (2015). The induction of pro-angiogenic processes within a collagen scaffold via exogenous estradiol and endometrial epithelial cells. Biotechnology and Bioengineering.

[94] GE Healthcare Life Sciences. Biacore Assay Handbook. *GE Healthcare Bio-Sciences AB* 1–78 (2012).

[95] Roden LD, Myszka DG (1996). Global analysis of a macromolecular interaction measured on BIAcore. Biochemical and biophysical research communications.

[96] Karlsson R, Fält A (1997). Experimental design for kinetic analysis of protein-protein interactions with surface plasmon resonance biosensors. Journal of immunological methods.

[97] Burke P, Schooler K, Wiley HS (2001). Regulation of epidermal growth factor receptor signaling by endocytosis and intracellular trafficking. Molecular biology of the cell.

[98] Sarabipour S, Ballmer-Hofer K, Hristova K (2016). VEGFR-2 conformational switch in response to ligand binding. eLife.

[99] Chen S, Guo X, Imarenezor O, Imoukhuede PI (2015). Quantification of VEGFRs, NRP1, and PDGFRs on Endothelial Cells and Fibroblasts Reveals Serum, Intra-Family Ligand, and Cross-Family LigandRegulation. Cellular and Molecular Bioengineering.

[100] Imoukhuede PI, Popel ASAS (2011). Quantification and cell-to-cell variation of vascular endothelial growth factor receptors. Experimental Cell Research.

[101] Imoukhuede PI, Popel AS (2014). Quantitative fluorescent profiling of VEGFRs reveals tumor cell and endothelial cell heterogeneity in breast cancer xenografts. Cancer medicine.

[102] Chen, S. *et al*. In *Biomedical Nanotechnology**:**Methods**and Protocol*s(eds. Petrosko, S. H. & Day, E. S.)117–138 (Springer New York, 2017). 10.1007/978-1-4939-6840-4_8.

[103] Papadopoulos N (2012). Binding and neutralization of vascular endothelial growth factor (VEGF) and related ligands by VEGF Trap, ranibizumab and bevacizumab. Angiogenesis.

[104] Finley, S. D., Chu, L.-H. & Popel, A. S. Computational systems biology approaches to anti-angiogenic cancer therapeutics. *Drug Discov Today* **0**, (2014). 10.1016/j.drudis.2014.09.026 PMC4336587 25286370

[105] Stefanini MO, Wu FTH, Mac Gabhann F, Popel AS (2010). Increase of plasma VEGF after intravenous administration of bevacizumab is predicted by a pharmacokinetic model. Cancer Research.

