# Author Correction: Discovery of High-Affinity PDGF-VEGFR Interactions: Redefining RTK Dynamics

**DOI:** 10.1038/s41598-020-63864-1

**Published:** 2020-06-30

**Authors:** Spencer B. Mamer, Si Chen, Jared C. Weddell, Alexandra Palasz, Ashley Wittenkeller, Manu Kumar, P. I. Imoukhuede

**Affiliations:** Department of Bioengineering, University of Illinois at Urbana-Champaign, Urbana, IL USA

Correction to: *Scientific Reports* 10.1038/s41598-017-16610-z, published online 27 November 2017

In this Article, in Figure 5, binding parameters for PDGF-CC, and -AB were incorrectly inputted into the plotting table. As a result of this some data points were misplaced. The correct Figure 5 appears below as Figure 1.

**Figure 1:**
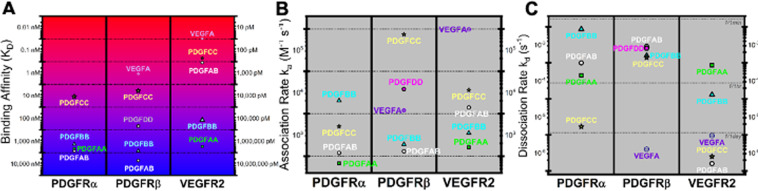
.

