# Supplementary material for: Discovery of High-Affinity PDGF-VEGFR Interactions: Redefining RTK Dynamics
Source: Sci Rep. 2017 Nov 27;7:16439. doi: 10.1038/s41598-017-16610-z (PMC5704011; doi:10.1038/s41598-017-16610-z)
Supplement: Supplementary file 1 — Supplementary File [file 41598_2017_16610_MOESM1_ESM.pdf]

Discovery of High-Affinity PDGF-VEGFR Interactions: Redefining RTK Dynamics

Authors: Spencer B. Mamer[1], Si Chen[1], Jared Weddell, Ph.D.[1], Alexandra Palasz[1], Ashley Wittenkeller[1], Manu Kumar[1], and P. I. Imoukhuede, Ph.D.[1*]

[1]Department of Bioengineering, University of Illinois at Urbana-Champaign, Urbana, IL
*Correspondence to: pii@illinois.edu

Supplementary Materials

Supplementary Tables

Supplementary Table S1. Classification of true and false interactions via χ^2-to-R_{max}. The ratio reflects how well a global kinetic analysis of a multi-concentration sensogram series can fit to a simple 1:1 Langmuir interaction model can fit. True interactions were distinguished from those due to non-specific binding patterns where χ^2-to-R_{max} < 1.0 (indicated by green shading and polka dot texturing). Fitted curves where χ^2-to-R_{max} > 1.0 (in red-shaded, grid-pattern textured cells) were considered to be dominated by non-specific binding. Ligand-receptor pairs that produced negative association curves were not analyzed with global fitting (indicated by "NI" for no interaction).

	VEGFR1	VEGFR2	VEGFR3	PDGFRα	PDGFRβ
**VEGFA**	0.17	0.52	6.03	34.4	0.95
**PDGFAA**	NI	0.082	NI	0.023	8.6
**PDGFAB**	NI	0.29	NI	0.0042	0.19
**PDGFBB**	NI	0.029	NI	0.0082	0.0064
**PDGFCC**	NI	0.33	NI	0.43	0.58
**PDGFDD**	NI	NI	NI	1130	2.2

Supplementary Table S2. VEGF and PDGF binding kinetics and affinities. Sensograms for ligand injections at 10 nM, 20 nM, and 40 nM, two replicates each, for each ligand-receptor pair. Kinetic constants were obtained by performing global kinetic analysis using the BIAevaluation software across several kinetic binding sensograms. Affinity constants were calculated as the ratio of the association and dissociation rates. Rate constants where the χ^2-to-R_{max} ratio exceeds 1.0 are shown *in italics*, indicating interactions that are dominated by non-specific interactions. Ligand-receptor pairs that produced negative association curves were not analyzed with global fitting (indicated by “NI” for no interaction). Mean values are reported for each kinetic fit for N=3. Uncertainty presented using the standard error of the mean.

	**VEGFR1**			**VEGFR2**			**VEGFR3**		
	**k_a** (M^{-1} s^{-1})	**k_d** (s^{-1})	**K_D** (M)	**k_a** (M^{-1} s^{-1})	**k_d** (s^{-1})	**K_D** (M)	**k_a** (M^{-1} s^{-1})	**k_d** (s^{-1})	**K_D** (M)
**VEGFA**	$4.0 \pm 0.04 * 10^{5}$	$4.0 \pm 0.1 * 10^{-7}$	$1.0 \pm 0.03 * 10^{-12}$	$9.7 \pm 0.3 * 10^{5}$	$9.5 \pm 0.2 * 10^{-6}$	$9.8 \pm 0.4 * 10^{-12}$	*2.2*	*$1.3 * 10^{-5}$*	*$5.2 * 10^{-6}$*
**PDGFAA**	NI	NI	NI	$1.6 \pm 0.1 * 10^{3}$	$8.6 \pm 0.1 * 10^{-4}$	$5.3 \pm 0.4 * 10^{-7}$	NI	NI	NI
**PDGFAB**	NI	NI	NI	$1.4 \pm 0.3 * 10^{3}$	$1.6 \pm 0.6 * 10^{-6}$	$1.1 \pm 0.5 * 10^{-10}$	NI	NI	NI
**PDGFBB**	NI	NI	NI	$3.5 \pm 0.7 * 10^{3}$	$1.3 \pm 0.1 * 10^{-4}$	$3.7 \pm 0.9 * 10^{-8}$	NI	NI	NI
**PDGFCC**	NI	NI	NI	$3.6 \pm 0.2 * 10^{4}$	$2.6 \pm 0.3 * 10^{-5}$	$7.0 \pm 0.9 * 10^{-11}$	NI	NI	NI
**PDGFDD**	NI	NI	NI	NI	NI	NI	NI	NI	NI

	**PDGFRα**			**PDGFRβ**		
	**k_a** (M^{-1} s^{-1})	**k_d** (s^{-1})	**K_D** (M)	**k_a** (M^{-1} s^{-1})	**k_d** (s^{-1})	**K_D** (M)
**VEGFA**	*$1.1 * 10^{3}$*	*$2.7 * 10^{-5}$*	*$2.6 * 10^{-8}$*	$1.2 \pm 0.1 * 10^{4}$	$4.0 \pm 0.5 * 10^{-6}$	$3.4 \pm 0.5 * 10^{-10}$
**PDGFAA**	$6.8 \pm 1.3 * 10^{2}$	$4.5 \pm 1.3 * 10^{-4}$	$6.6 \pm 2.3 * 10^{-7}$	*$8.8 \pm 0.4 * 10^{3}$*	*$1.5 \pm 0.4 * 10^{-4}$*	*$1.7 \pm 0.4 * 10^{-8}$*
**PDGFAB**	$1.2 \pm 0.1 * 10^{3}$	$1.0 \pm 0.2 * 10^{-3}$	$8.4 \pm 1.7 * 10^{-7}$	$1.3 \pm 0.3 * 10^{3}$	$2.9 \pm 0.7 * 10^{-3}$	$2.2 \pm 0.7 * 10^{-6}$
**PDGFBB**	$2.0 \pm 0.1 * 10^{4}$	$8.6 \pm 0.1 * 10^{-3}$	$4.2 \pm 0.3 * 10^{-7}$	$1.9 \pm 0.1 * 10^{3}$	$1.6 \pm 0.2 * 10^{-3}$	$8.3 \pm 1.3 * 10^{-7}$
**PDGFCC**	$5.0 \pm 1.6 * 10^{3}$	$2.0 \pm 0.1 * 10^{-4}$	$3.4 \pm 1.8 * 10^{-9}$	$7.4 \pm 0.4 * 10^{5}$	$1.4 \pm 0.06 * 10^{-3}$	$1.9 \pm 0.1 * 10^{-9}$
**PDGFDD**	*18.8*	*$1.0 * 10^{-5}$*	*$5.4 * 10^{-7}$*	*$3.8 * 10^{4}$*	*$2.6 * 10^{-3}$*	*$6.7 * 10^{-8}$*

Supplementary Table S3. Literature mined VEGF and PDGF from SPR and cell-based assays. Values determined with cell assays indicated with † and SPR indicated with an asterisk***.** Currently, few interaction kinetics have been determined.

Interaction	Measured K_D (M)
**VEGF-A:VEGFR1**	$7.4*10^{-11}$ ***** - $7.5*10^{-12}$ M ([1])†
**VEGF-A:VEGFR2**	$5.2*10^{-11}$ M ([1]) †
**PDGF-AA:PDGFRα**	$1.34*10^{-8}$ M ([2])*****
**PDGF-AA:PDGFRβ**	$4.53*10^{-7}$ M ([2])*****
**PDGF-BB:PDGFRα**	$1.5*10^{-7}$ M ([2])*****
**PDGF-BB:PDGFRβ**	$1.6*10^{-9}$ M ([2])*****

Supplementary Table S4. Model kinetic parameters and initial values.

	Benchmark	Healthy	Cancer	Units
**VEGFA:VEGFR1 coupling**				
$k_{1,f}$ (coupling)	$3.8 * 10^{6}$ (3)	$4.0 * 10^{5}$ (via SPR)		$M^{-1} s^{-1}$
$k_{1,r}$ (decoupling)	$95 * 10^{-6}$ (3)	$4.0 * 10^{-7}$ (via SPR)		s^{-1}
**VEGFA:VEGFR2 coupling**				
$k_{2,f}$ (coupling)	$1.2 * 10^{6}$ (3)	$9.7 * 10^{5}$ (via SPR)		$M^{-1} s^{-1}$
$K_{2,r}$ (decoupling)	$410 * 10^{-6}$ (3)	$9.5 * 10^{-6}$ (via SPR)		s^{-1}
**PDGF:VEGFR2 coupling**	*N/A*	See **Supplementary Table S1**		
**Receptor and ligand-receptor complex internalization**				
k_{int}	10^{-5} (3)			s^{-1}
**Receptor insertion**				
$k_{insert,R1}$	0.8 (3)			s^{-1}
$k_{insert,R2}$	2.3 (3)			s^{-1}
**Extracellular volume**				
V_{mem}	10^{-10} (4)			L
**VEGFA-bevacizumab coupling**				
$k_{7,f}$	4.10×10^{7} (5)	$5.3 * 10^{5}$ (6)		$M^{-1} s^{-1}$
$k_{7,r}$	2.01×10^{-5} (5)	$3.1 * 10^{-5}$ (6)		s^{-1}
**VEGFR1 concentration/EC**				
D_{11}	80,000 (3)	990 (7)	8200 (8)	receptors
**VEGFR2 concentration/EC**				
D_{22}	230,000 (3)	1890 (7)	1100 (8)	receptors
**Healthy serum concentrations**				
VA	2.2 (3)	See **Supplementary Table S3**		nM
P_{AA}	N/A			nM
P_{AB}	N/A			nM
P_{BB}	N/A			nM
P_{CC}	N/A			nM

Supplementary Table S5. Serum concentrations for VEGF-A and PDGFs under physiological and pathological conditions. All values are means (± SD where provided) unless otherwise noted.

	[VEGF-A]	[PDGF-AA]	[PDGF-AB]	[PDGF-BB]	[PDGF-CC]	[PDGF-DD]
**Normal (serum)**	81.7-91.83 pg/m[9,10]	250.0-1700 pg/mL[10–12]	250.0-2830 pg/mL[11]	8506 ± 550 pg/mL[13]	None	1170 ± 460 pg/mL[14]
**Exercise (serum)**	165.61 pg/mL[10]	4640 pg/mL[10]				
**Wound fluid**	1033 pg/mL[15]	8604 pg/mL[15]				
**Stroke (0hr) (serum)**	410 ± 71 pg/mL[16]	7300 ± 5100 pg/mL[17]	52600 ± 18900 pg/mL[17]	10100 ± 7700 pg/mL[17]	66900 ± 41200 pg/mL[17]	
**Stroke (24hr) (serum)**	416 ± 64 pg/mL[16]	5500 ± 3900 pg/mL[17]	48000 ± 16400 pg/mL[17]	7000 ± 4700 pg/mL[17]	123600 ± 64000 pg/mL[17]	
**Breast Cancer (serum)**	305.9 pg/mL[18]	11900 ± 5100 pg/mL[19]	11900 ± 5100 pg/mL[19]	7623 pg/mL[20]		7500 pg/mL[21]

Supplementary Table S6. Isoelectric points of immobilized proteins and optimal immobilization pHs.

Protein	pI	Optimal pH
**VEGFR1**	8.7[22]	3.7
**VEGFR2**	5.6[23]	4.0
**VEGFR3**	5.9[24]	5.0
**PDGFRα**	5.0[25]	4.5
**PDGFRβ**	4.9[26]	3.7
**Angiopoietin-4**	9.1[27]	4.0

References

1. von Tiedemann, B. & Bilitewski, U. Characterization of the vascular endothelial growth factor-receptor interaction and determination of the recombinant protein by an optical receptor sensor. *Biosensors and Bioelectronics* **17,** 983–991 (2002).
2. Lin, X., Takahashi, K., Liu, Y., Derrien, A. & Zamora, P. O. A synthetic, bioactive PDGF mimetic with binding to both alpha-PDGF and beta-PDGF receptors. *Growth factors (Chur, Switzerland)* **25,** 87–93 (2007).
3. Mac Gabhann, F. *et al.* Model of competitive binding of vascular endothelial growth factor and placental growth factor to VEGF receptors on endothelial cells. *American journal of physiology. Heart and circulatory physiology* **286,** H153-64 (2004).
4. Wei, D. *et al.* High-resolution three-dimensional reconstruction of a whole yeast cell using focused-ion beam scanning electron microscopy. *BioTechniques* **53,** 41–48 (2012).
5. Finley, S. D. & Popel, A. S. Predicting the effects of anti-angiogenic agents targeting specific VEGF isoforms. *The AAPS journal* **14,** 500–9 (2012).
6. Papadopoulos, N. *et al.* Binding and neutralization of vascular endothelial growth factor (VEGF) and related ligands by VEGF Trap, ranibizumab and bevacizumab. *Angiogenesis* **15,** 171–185 (2012).
7. Chen, S., Guo, X., Imarenezor, O. & Imoukhuede, P. I. Quantification of VEGFRs, NRP1, and PDGFRs on Endothelial Cells and Fibroblasts Reveals Serum, Intra-Family Ligand, and Cross-Family Ligand Regulation. *Cellular and Molecular Bioengineering* **8,** 383–403 (2015).
8. Imoukhuede, P. I. & Popel, A. S. Quantitative fluorescent profiling of VEGFRs reveals tumor cell and endothelial cell heterogeneity in breast cancer xenografts. *Cancer medicine* **3,** 225–244 (2014).
9. Soeki, T. *et al.* Serial changes in serum VEGF and HGF in patients with acute myocardial infarction. *Cardiology* **93,** 168–174 (2000).
10. Czarkowska-Paczek, B., Bartlomiejczyk, I. & Przybylski, J. The serum levels of growth factors: PDGF, TGF-beta and VEGF are increased after strenuous physical exercise. *Journal of Physiology and Pharmacology* **57,** 189–197 (2006).
11. Harrison, A. A., Dunbar, P. R. & Neale, T. J. Immunoassay of platelet-derived growth factor in the blood of patients with diabetes mellitus. *Diabetologia* **37,** 1142–1146 (1997).
12. Nowak, M. *et al.* Serum Concentration of VEGF and PDGF-AA in Patients with Active Thyroid Orbitopathy before and after Immunosuppressive Therapy. *Exp Clin Endocrinol Diabetes* **122,** 582–586 (2014).
13. Takayama, H. *et al.* Serum levels of platelet-derived growth factor-BB and vascular endothelial growth factor as prognostic factors for patients with fulminant hepatic failure. *Journal of gastroenterology and hepatology* **26,** 116–121 (2011).
14. Bolouri, H. Network dynamics in the tumor microenvironment. *Seminars in Cancer Biology* **30,** 52–59 (2015).
15. Baker, E. A. & Leaper, D. J. Proteinases, their inhibitors, and cytokine profiles in acute wound fluid. *Wound Repair and Regeneration* **8,** 392–398 (2000).
16. Gunsilius, E., Petzer, A. L., Stockhammer, G., Kähler, C. M. & Gastl, G. Serial measurement of vascular endothelial growth factor and transforming growth

factor-beta1 in serum of patients with acute ischemic stroke. *Stroke; a journal of cerebral circulation* **32,** 275–278 (2001).
17. Rodríguez-González, R. *et al.* Platelet derived growth factor-CC isoform is associated with hemorrhagic transformation in ischemic stroke patients treated with tissue plasminogen activator. *Atherosclerosis* **226,** 165–171 (2013).
18. Heer, K. *et al.* Serum Vascular Endothelial Growth Factor in Breast Cancer: Its Relation with Cancer Type and Estrogen Receptor Status . *Clinical Cancer Research* **7,** 3491–3494 (2001).
19. Pasanisi, P. *et al.* Serum Insulin-Like Growth Factor-I and Platelet-Derived Growth Factor as Biomarkers of Breast Cancer Prognosis. *Cancer epidemiology, biomarkers & prevention : a publication of the American Association for Cancer Research, cosponsored by the American Society of Preventive Oncology* **17,** 1719–1722 (2008).
20. Rykala, J. *et al.* Angiogenesis markers quantification in breast cancer and their correlation with clinicopathological prognostic variables. *Pathology and Oncology Research* **17,** 809–817 (2011).
21. LaRochelle, W. J. *et al.* Platelet-derived Growth Factor D: Tumorigenicity in Mice and Dysregulated Expression in Human Cancer. *Cancer Research* **62,** 2468 LP-2473 (2002).
22. VEGFR1 (human). *PhosphoSitePlus* **2014,**
23. VEGFR2 (human). *PhosphoSitePlus* **2014,**
24. VEGFR3 (human). *PhosphoSitePlus* **2014,**
25. PDGFRA (human). *PhosphoSitePlus* **2014,**
26. PDGFRB (human). *PhosphoSitePlus* **2014,**
27. ANGPT4 (human). **2014,**

Supplementary Figures

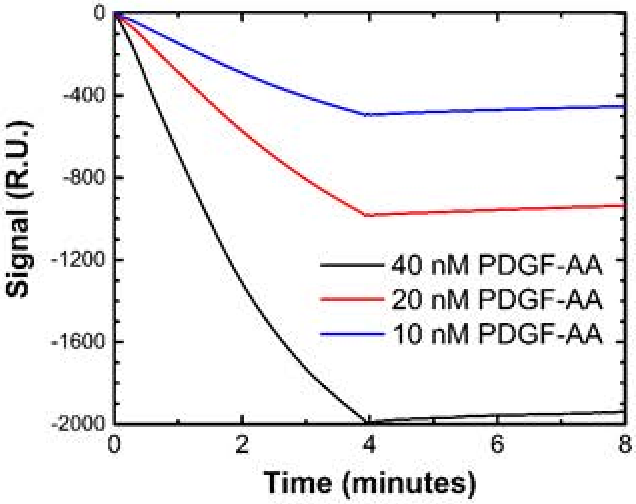

Supplementary Figure S1. Non-specific signal example--PDGF-AA: VEGFR3. Sensogram depicts example negative reference-subtracted curve produced when subtracting Ang-4 reference signal from binding signal. The negative response signal suggests than non-specific binding effects are greater than specific, 'true' binding effects.

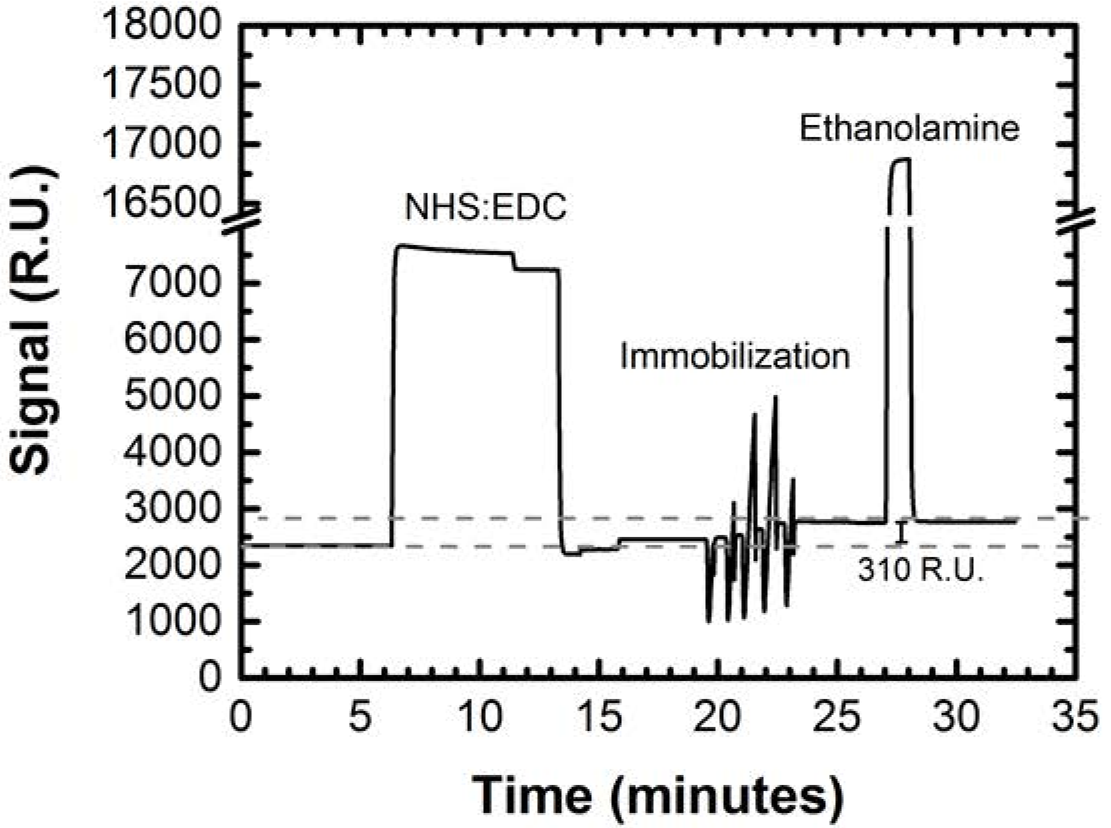

Supplementary Figure S2. SPR immobilization example—PDGFRα binding to the dextran matrix. Surface activated by injecting 35 μL 1:1 NHS:EDC solution at flow rate of 5 μL min^{-1}. Injected 20 μg mL^{-1} PDGFRα in 10mM pH 3.7 acetate buffer until 310 R.U. of receptor immobilized. Surface deactivated by injecting 5 μL ethanolamine-HCL at 5 μL min^{-1}.

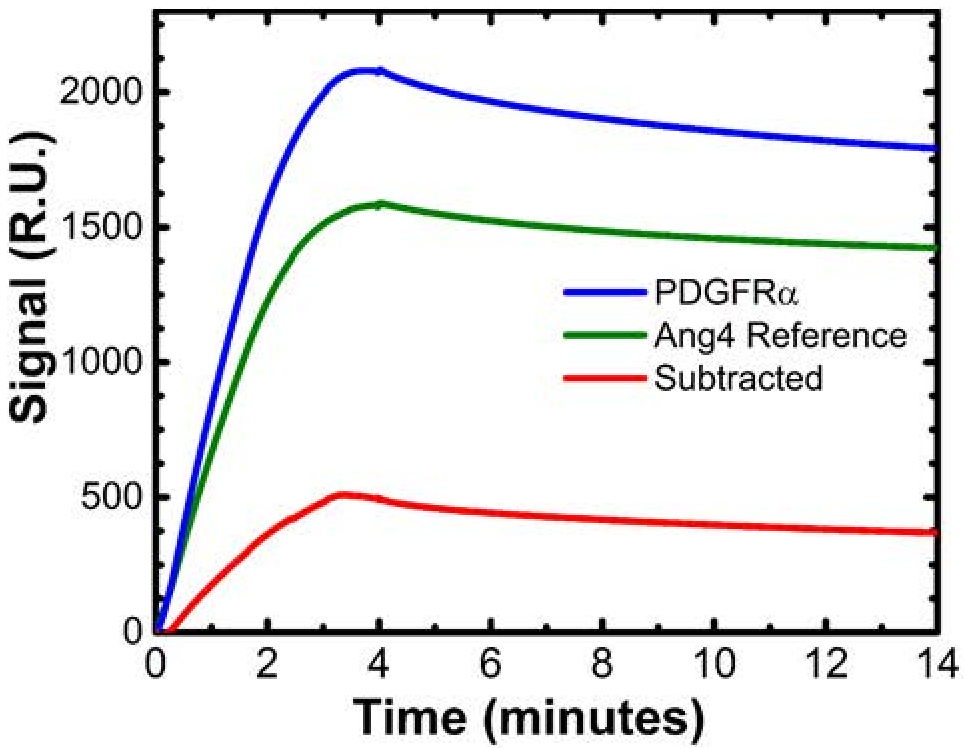

Supplementary Figure S3. Non-specific binding reference signal subtraction. Sensogram depicts the injection of 40 nM PDGFAA across immobilized angiopoietin-4 and PDGFRα simultaneously. Ang-4, a protein with no known interaction with either PDGF or VEGF ligands, was immobilized at levels similar to the receptors as a background reference signal. The reference subtracted curve was obtained by subtracting this reference signal from the raw receptor-ligand interaction curve, removing signals associated with non-specific interactions.
